# Evaluation of a Room-Temperature Preservation Method Maintaining Viability and Function in Human Cardiac Organoids

**DOI:** 10.3390/cells15121065

**Published:** 2026-06-11

**Authors:** Cynthia Van Rompay, Kevin Tabury, Emil Rehnberg, Zoë Janssen, Sarah Baatout, Marianne S. Carlon, Xavier Casadevall i Solvas, Bjorn Baselet

**Affiliations:** 1 Nuclear Medical Applications, Radiobiology Unit, Belgian Nuclear Research Centre SCK CEN, 2400 Mol, Belgium; cynthia.van.rompay@sckcen.be (C.V.R.); emil.rehnberg@ugent.be (E.R.); zoe.janssen@sckcen.be (Z.J.); sarah.baatout@sckcen.be (S.B.); 2 Biomimetics Group, Department of Biosystems, Division Mechatronics, Biostatistics and Sensors (MeBioS), KU Leuven, 3001 Leuven, Belgium; xevi.casadevall@kuleuven.be; 3 Department of Biomedical Engineering, College of Engineering and Computing, University of South Carolina, Columbia, SC 29208, USA; 4 Department of Physics and Astronomy, Faculty of Sciences, Ghent University, 9000 Ghent, Belgium; 5 Laboratory of Respiratory Diseases and Thoracic Surgery (BREATHE), Department of Chronic Diseases and Metabolism, KU Leuven, 3000 Leuven, Belgium; marianne.carlon@kuleuven.be

**Keywords:** 3D cardiac models, spheroids, organoids, cryopreservation, room temperature-based preservation

## Abstract

Three-dimensional (3D) cardiac models, including spheroids, organoids, and organ-on-chips, are advanced systems for studying human physiology, disease, and drug responses with greater biological relevance than 2D models. As their use expands in biomedical research, tissue engineering, and regenerative medicine, reliable preservation methods are needed. However, cryopreservation often fails to protect 3D systems due to limited cryoprotectant penetration, ice formation, and mechanical stress during freezing and thawing. Room-temperature (RT) preservation has emerged as a promising alternative for short-term transport. This study evaluated a RT-based transport medium (CellShip^®^) for preserving cardiac organoids for up to seven days, compared with conventional cryopreservation using slow-freezing in Cryostor^®^CS10. Viability and functionality were assessed using apoptosis, ATP levels, beating activity, proliferation, and size. During maturation, organoids showed increased size, ATP levels, and beating capacity. Cryopreservation reduced size, proliferation, ATP levels, and altered beating, while increasing apoptosis. In contrast, RT preservation maintained stable viability and functionality after recovery. These findings demonstrate that RT preservation effectively maintains cardiac organoid integrity and function, offering a promising alternative for short-term storage and transport, with potential terrestrial and nonterrestrial applications.

## 1. Introduction

Complex three-dimensional (3D) cardiac models, including spheroids, organoids and organ-on-chips, have emerged as powerful molecular systems to study human physiology, disease mechanisms and drug responses more accurately than conventional two-dimensional (2D) cellular models do [1,2]. These 3D cardiac models more closely recapitulate the in vivo anatomy, cell composition and physiology of human tissues, including cell–cell interactions, oxygen and nutrient gradients, and extracellular matrix (ECM) dynamics [1,2,3]. As their application has gained traction in the fields of biomedical research, tissue engineering and regenerative medicine, a reliable and scalable preservation method would strengthen the use of these models [3,4]. Today, conventional cryopreservation is applied as a standard method to preserve cells and biological material. This approach offers scalability, reproducibility and global accessibility while minimizing the loss of viability and functional properties [5,6,7]. However, conventional cryopreservation often fails to protect complex 3D cellular systems because of their structural multicellular organization, limited penetration of cryoprotective agents (CPAs) and CPA-associated toxicity. Insufficient CPA diffusion into dense 3D constructs can lead to ice crystal formation and osmotic stress during freezing and thawing, resulting in cellular damage and functional impairment [5,8,9,10,11,12]. Ice crystal formation can be reduced by ultrarapid cooling approaches, such as vitrification, where high CPA concentrations are used, resulting in improved preservation outcomes including enhanced cell viability, when these methods are applied to sensitive models including stem cell-derived tissues or organoids [4,9,11,13]. Nevertheless, high-throughput vitrification of larger tissues is rather limited due to the toxicity risks associated with high CPA concentrations [11,14].

Microfluidic perfusion devices can gradually deliver CPAs and uniformly remove them upon thawing through microfluidic channels, thereby providing a solution to the problem of CPA toxicity, improving CPA penetration through the channels or potentially even eliminating the need for CPAs, thus reducing osmotic shock and toxicity [15,16,17,18,19]. Although it supports cryopreservation with limited or no use of toxic CPAs, this preservation strategy is still in an early stage of development, resulting in a lack of standardization, low throughput and the need for specialized fabrication and devices [15].

To further overcome these limitations, alternative preservation technologies have recently emerged, including the use of magnetic nanoparticles in so-called nanowarming. This technology offers an alternative for larger cellular constructs or tissues to rapidly freeze and rewarm vitrified tissues with uniform thermal gradients, thereby reducing ice recrystallization during the warming process [20,21,22,23,24,25,26]. As a more suitable preservation method for sensitive models such as organoids, hydrogel-based cryopreservation is often used to maintain the ECM structure and intracellular organization. Here, organoids or tissues are encapsulated in hydrogels (e.g., alginate or polyethylene glycol) to provide mechanical support during freezing and protect against deformation caused by ice expansion [5,8,12,20,27]. However, the hydrogel composition may alter cell behavior and restrict the broader applicability of this strategy [28,29]. Additionally, successful attempts have been made to preserve cells under hypothermic conditions to eliminate the freezer and liquid nitrogen requirements [5,8,11,30,31,32,33]. However, this strategy still requires significant optimization to maintain ECM and cell viability and is therefore still experimental for complex 3D models. Finally, room-temperature-based preservation strategies for single cells have been explored across different cell lines, offering a new paradigm of opportunities [34]. Overall, a reliable and scalable preservation strategy for 3D in vitro models that limits or even eliminates ice crystal formation, CPA toxicity, ultralow-temperature freezing requirements and the need for advanced equipment has not yet been established. These findings indicate the growing potential for reliable and standardized preservation methodologies for 3D cellular models, including cardiac organoids.

The development of preservation strategies for 3D in vitro models that are compatible with high-throughput screening and biobanking could drive innovations in tissue and organ preservation and, when improved in scaling, advance organ transplantation. The growing shortage of available organs would diminish by extending the lifetime of donated organs, and thus extending the time needed to find a suitable recipient [35,36]. Furthermore, organ slices are valuable experimental platforms that can preserve native tissue architecture, multicellular interactions, and physiological functionality [37,38,39,40,41]. Alongside healthy tissue models, diseased organoids and pathological tissue constructs are also gaining importance for studying disease mechanisms and evaluating therapeutic interventions [37,40,42,43]. However, the widespread application and distribution of these complex models remain challenged by the lack of efficient and reliable preservation strategies. Importantly, diseased tissues may exhibit altered metabolic activity, ECM organization, oxygen consumption, and stress sensitivity compared to healthy tissues, potentially affecting their preservation and recovery capacity [44,45,46,47]. Therefore, preservation methods require careful optimization and validation depending on the tissue type, structural complexity, and disease context to ensure maintenance of tissue integrity, viability, and functionality after recovery.

In addition to the application on Earth, human cell-derived 3D in vitro models are increasingly used in space research to mimic the adverse health effects experienced by astronauts. Investigating the harmful effects of space travel on the human body is crucial for performing health risk assessments of astronauts during future space missions [48,49,50,51,52]. The development of preservation technologies that maintain both the viability and functional integrity of 3D cellular models during space travel could therefore help address important logistical constraints and enable new applications in biological space research.

In this study, we evaluated two preservation approaches suitable for 3D cardiac organoids, slow-freezing cryopreservation followed by storage in liquid nitrogen, and room-temperature-based preservation, to determine their ability to maintain cardioid viability, morphology and functional properties after recovery.

## 2. Materials and Methods

### 2.1. Cell Culture

#### 2.1.1. Organoids

Cardiac organoids were generated from human induced pluripotent stem cells (hiPSCs) (UCSD163i-95-1, WiCell Research Institute, Madison, WI, USA) and maintained at 37 °C in a humidified atmosphere containing 95% air and 5% CO_2_. Cardiomyocyte differentiation was performed based on the differentiation protocol previously published by Hofbauer et al. [53] with adaptations. This eight-day differentiation protocol includes mesoderm induction, cardiac mesoderm induction and cardiomyocyte specification whereafter cardiac organoids are reassociated into a Corning^®^ 96-well black/clear round bottom ultralow attachment surface spheroid microplate (Corning, 4520, Berlin, Germany) (indicated as day 0 in our study design). Cardiac organoids were maintained in maintenance medium, chemically defined medium (CDM) prepared by mixing 50% IMDM (GIBCO^TM^, 12440053, Thermo-Fischer Scientific, Waltham, MA, USA) and 50% F-12 Nutrient Mix (GIBCO^TM^, 11765054, Thermo-Fischer Scientific, Waltham, MA, USA) and adding 5 mg/mL BSA (Sigma-Aldrich, A7030, Merck KGaA, Darmstadt, Germany), 1% chemically defined concentrated lipids (GIBCO^TM^, 11905031, Thermo-Fischer Scientific, Waltham, MA, USA), 0.004% 1-thiolglycerol (Sigma-Aldrich, M6145, Merck KGaA, Darmstadt, Germany) and 15 µg/mL Transferrin (Sigma-Aldrich, 178481, Merck KGaA, Darmstadt, Germany) with subsequent 0.2 µm filter filtration. This CDM was supplemented with 10 µg/mL insulin (Roche, 11376497001, Merck KGaA, Darmstadt, Germany) and 100 ng/mL VEGF-165 (STEMCELL, 78159.1, Saint Égrève, France). Size measurements based on the cardioid diameter were performed via brightfield images taken with the Leica Application Suite microscope software V4.13 platform (Leica Microsystems, Wetzlar, Germany).

#### 2.1.2. Treatments

Cardiac models were treated with staurosporine (1 µM stock, Sigma-Aldrich, S6942, Merck KGaA, Darmstadt, Germany) to induce apoptosis as a positive control for cell death measurements. The treated models were incubated for 24 h at 37 °C in a humidified atmosphere containing CO_2_.

### 2.2. Preservation Method

#### 2.2.1. Cryopreservation and Thawing

For cryopreservation, ten-days old cardiac organoids were resuspended in cryopreservation medium including Cryostor^®^CS10 (STEMCELL^TM^ Technologies, 100-1061, Saint Égrève, France). Standard cryopreservation following a slow-freezing protocol was initiated by transferring the 3D cardiac constructs into a cryovial (Cryo.S, 2 mL, round bottom, Greiner Bio-One, Vilvoorde, Belgium) by adding four organoids per cryovial with 1 mL cryopreservation medium, with subsequent cooling and storage overnight in a CoolCell^TM^ freezing vial container at −80 °C to ensure a slow cooling rate of −1 °C min^−1^. After overnight incubation, the cryovials were transferred to a liquid nitrogen freezer (−196 °C), where they remained for an additional seven or fourteen days. Post preservation, the samples were thawed in a 37 °C water bath until only a small ice clump remained, after which the cardiac constructs were transferred to a Corning^®^ 96-well Black/Clear Round Bottom Ultra-Low Attachment Surface Spheroid Microplate (Corning, 4520, Berlin, Germany) after washing with prewarmed maintenance medium supplemented with 10 µM ROCK inhibitor Y-27632 (STEMCELL, 72304, Saint Égrève, France). After 24 h, the medium was changed to standard maintenance medium. One day (24 h) and one week (1 week) post thawing, viability and functionality measurements were performed. Unpreserved controls were maintained at 37 °C in a humidified atmosphere containing 95% air and 5% CO_2_. Controls were collected after the same total duration of cell culture as the preserved samples, but without undergoing preservation. Specifically, cultures at day 10 + 24 h served as controls for samples preserved for 7 or 14 days followed by 24 h of recovery, whereas cultures at day 10 + 1 week served as controls for samples preserved for 7 or 14 days followed by 1 week of recovery.

#### 2.2.2. Room Temperature-Based Preservation

Ten-day old cardiac organoids were transferred to a Corning^®^ 96-well Black/Clear Round Bottom Ultra-Low Attachment Surface Spheroid Microplate (Corning, 4520, Berlin, Germany), containing 200 µL of CellShip^®^ Cell Transport medium without insulin (Life Science Production, SCS-004K, Bedfordshire, UK) and a single cardioid organoid per well. These plates were sealed using Nunc™ Seals (Thermo-Fisher Scientific, 236366, Waltham, MA, USA) to prevent medium evaporation and stored in a transport box to prevent vibrations for preservation at room temperature (20–25 °C). After seven or fourteen days of preservation, the cardiac constructs were transferred to a Corning^®^ 96-well Black/Clear Round Bottom Ultra-Low Attachment Surface Spheroid Microplate (Corning, 4520, Berlin, Germany) with prewarmed maintenance medium supplemented with 10 µM ROCK inhibitor Y-27632 (STEMCELL, 72304, Saint Égrève, France). After 24 h, the medium was changed to standard maintenance medium. One day (24 h), one week and three weeks post-preservation, viability and functionality measurements were performed. Unpreserved controls were maintained at 37 °C in a humidified atmosphere containing 95% air and 5% CO_2_. Controls were collected after the same total duration of cell culture as the preserved samples, but without undergoing preservation. Specifically, cultures at day 10 + 24 h served as controls for preserved samples with 24 h of recovery, cultures at day 10 + 1 week served as controls for preserved samples with 1 week of recovery, and cultures at day 10 + 3 weeks served as controls for preserved samples with 3 weeks of recovery.

### 2.3. Immunofluorescence Imaging

#### 2.3.1. Cryosections

Cardiac models were fixed with 10% neutral buffered formalin solution (CellStor, CellPath, Newtown Powys, UK) at room temperature (RT) before being immersed in 30% sucrose solution until use, followed by embedding in cryogel for cryosectioning at 10 µm using a cryostat (MICROM International GmbH, part of Thermo Scientific CryoStar NX50, Walldorf, Germany). The sections were mounted onto SuperFrost Plus Adhesion microscope slides (Epredia^TM^, 22-042-941,Thermo Fischer Scientific, Waltham, MA, USA).

#### 2.3.2. Immunohistochemistry

The cryosections were air-dried for 30 min, washed (3 × 5 min) with phosphate-buffered saline (PBS, Gibco), boiled in pH 6 citrate buffer for antigen retrieval (Agilent, S1699, Santa Clara, CA, USA), and washed in Tris-buffered saline with 0.1% Tween-20 (TBS-T, Sigma Aldrich, St. Louis, MO, USA) for permeabilization. Afterwards, the cells were blocked with 20% preimmune goat serum (PIG, Thermo-Fisher Scientific, 31872, Waltham, MA, USA) in Tris-NaCl-blocking buffer (TNB) for one hour at RT. Afterwards, the samples were incubated overnight at 4 °C with rabbit anti-cleaved caspase-3 (CC3) (1:200, Cell Signalling, 9661, Leiden, The Netherlands), rat anti-KI-67 (Antigen Kiel 67 (Ki67)) (1:1000, eBioscience™, 14-5698-82, Thermo Fischer Scientific, Waltham, MA, USA) and mouse anti-cardiac troponin T (CTNT) (1:, Thermo-Fischer Scientific, MA5-12960, Waltham, MA, USA) diluted in 5% PIG in TNB. After being washed with TBS-T (3 × 5 min), the cells were incubated with secondary goat anti-rat Alexa Fluor 568 (Invitrogen^TM^, Carlsbad, CA, USA, A-11077, diluted 1:200 in TNB, Thermo Fischer Scientific, Waltham, MA, USA), goat anti-rabbit Alexa Fluor 647 Plus (Invitrogen^TM^, A32733, diluted 1:1000 in TNB, Thermo Fischer Scientific, Waltham, MA, USA) and goat anti-mouse Alexa Fluor 568 (Invitrogen^TM^, A11031, diluted 1:1000 in TNB, Thermo Fischer Scientific, Waltham, MA, USA). The cells were then washed with TBS-T followed by incubation (15 min) with 4′,6-diamidino-2-phenylindole (DAPI, 5 µg/mL in PBS; Sigma-Aldrich, D9542, Merck KGaA, Darmstadt, Germany). Subsequently, the cryosections were mounted with Molecular Probes™ ProLong™ Diamond Antifade Mountant with DAPI (Thermo Fischer Scientific, 15810083, Waltham, MA, USA) and stored at −20 °C. A Nikon Eclipse Ti-E inverted widefield microscope (Nikon Instruments, Amstelveen, The Netherlands) with a 20× objective connected to a Prime BSI sCMOS camera was used to visualize cells in the cardiac models that were positive for the distinct markers. ImageJ version 1.54 was used to analyze the number of cells positive for cleaved caspase-3 or Ki67 based on intensity measurements with rolling ball correction via a nuclear mask on the DAPI signal and normalized to the total amount of nuclei.

### 2.4. Adenosine Triphosphate (ATP)-Level Quantification

The cardiac models were transferred to white opaque 96-well plates (F-bottom) (Greiner, 655083, Vilvoorde, Belgium) with standard culture medium added to the wells. One single organoid was transferred to a single well in the 96-well plate to maintain equal organoid concentration per condition. An equal volume of the CellTiter-Glo^®^ 3D reagent from the CellTiter-Glo^®^ 3D Cell Viability Assay (Promega, G9682, Leiden, The Netherlands) was added. The contents were vigorously mixed for 5 min on a plate shaker to induce cell lysis and incubated for an additional 25 min to stabilize the luminescent signal. Luminescence was recorded via a CLARIOstar Plus Microplate Reader (BMG LABTECH, Ortenberg, Germany).

### 2.5. Assessment of Functional Beating

Real-time videos of beating cardiac organoids were recorded as .AVI files via a Nikon Eclipse Ti-E inverted microscope (Nikon Instruments, The Netherlands) at 37 °C with a 5× objective (rate: 50 fps, duration: 30 s, no compression). The open-source software tool MUSCLEMOTION V1.0 [54] was used to quantify cardioid contraction profiles on the basis of various parameters including contraction duration, relaxation time, peak-to-peak time, and 10-to-10 transient time in milliseconds (ms). The means with standard deviations were included in the individual graphs for each individual parameter.

### 2.6. Statistical Analyses

Statistical analyses were performed in GraphPad Prism version 10.4.0 for Windows (GraphPad Software, San Diego, CA, USA, www.graphpad.com). The results represent the average of two-to-three biological replicates, each of which included cardiac models per condition ± standard deviation (SD). For each endpoint, outliers were identified via Grubb’s test, which is based on a test statistic, that is calculated from the most extreme data point and corresponds to a *p* value that represents the likelihood of detecting the outlier. The data were first tested for normality and homoscedasticity. To test for significant group differences, two-way ANOVA was performed if the data were normally distributed with equal variances, otherwise the Kruskal-Wallis test was used as nonparametric alternative. The Welch and Brown-Forsythe ANOVA test was used to compare three or more sets of unpaired measurements, which were assumed to be sampled from a Gaussian distribution but without assuming that the groups had equal variances. *p* values were corrected for multiple comparisons via Tukey’s multiple comparisons test, Dunn’s test or Dunnett’s test according to the statistical test. Correlation analysis was performed using the Pearson correlation test (95% confidence interval). Statistical significance was assumed when *p* values were <0.05.

## 3. Results

### 3.1. 3D Cardiac Model Validation

Two different 3D cellular models were initially used to evaluate the preservation methods. As a first model, cardiac spheroids were generated from immortalized cell lines including cardiomyocytes (AC16), cardiac fibroblasts (IM-HCF) and endothelial cells (TICAE). Samples were collected at 3, 7 and 14 days after spheroid generation to evaluate viability and functionality over time during spheroid maturation (Figures S1 and S2). The first samples were collected on day 3 after seeding to allow the cells to form spheroids that were compact enough to tolerate manipulation during sample collection. Overall, spheroid size, adenosine triphosphate (ATP) levels and the number of proliferative (Ki67-positive) cells decreased after one week of culture (Figures S1 and S2). Together with increasing numbers of apoptotic (CC3-positive) cells measured over time, these results suggest that the viability of our cardiac spheroids diminishes over time during maturation (Figures S1 and S2). Therefore, a next cardiac model was introduced that provides the implementation of an extra readout, namely, the beating capacity, which allows real-time monitoring of functional beating.

**Figure 1:**
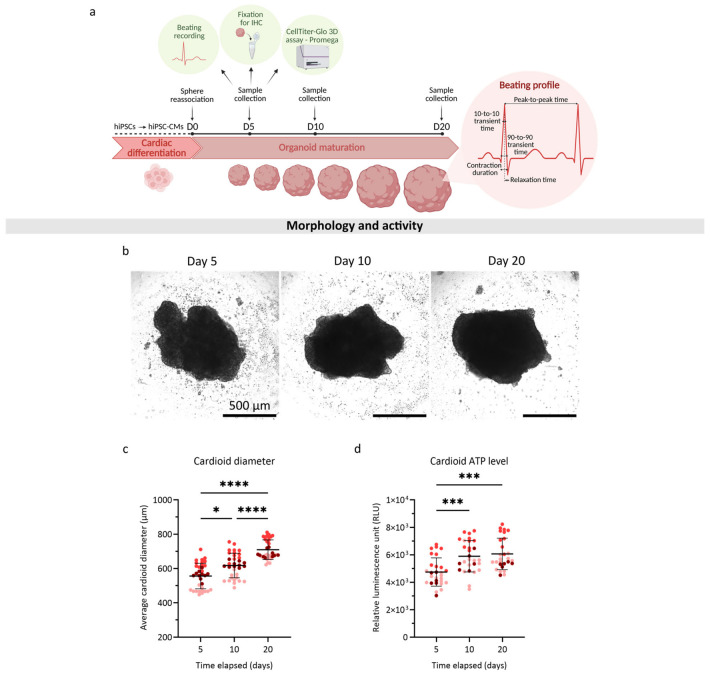
Cardiac organoid validation by baseline viability and functionality measurements. (**a**) Overview of the cardioid baseline experiment. D: day, hiPSCs: human induced pluripotent stem cells, CM: cardiomyocytes. Created in BioRender. Bijnens, M. (2026) https://BioRender.com/mbejtnw. (**b**) Brightfield images of cardiac cardioids over time during maturation. Scale bar: 500 µm. (**c**) Average cardioid diameter over time during maturation. Shades of colors represent biological replicates (N = 3). The data are presented as the means ± SD, and the Kruskal–Wallis test with Dunn’s multiple comparisons test was used. * *p* < 0.05; **** *p* < 0.001. (**d**) Averaged cardioid ATP levels over time during maturation. ATP: adenosine triphosphate. Shades represent biological replicates (N = 3). The data are presented as the means ± SD. The Kruskal–Wallis test with Dunn’s multiple comparisons test was used. *** *p* < 0.005.

**Figure 2:**
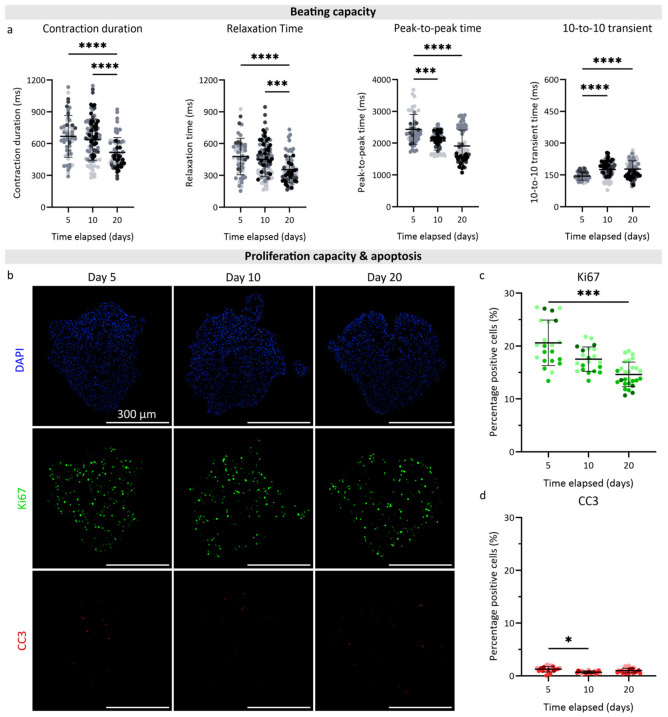
Cardiac organoid beating, proliferation capacity and apoptosis. (**a**) Beating parameters of cardiac organoids: contraction duration, relaxation time, peak-to-peak time, and 10-to-10 transient time in milliseconds (ms). Shades of colors represent biological replicates (N = 3). The data are presented as the means ± SD. The Kruskal–Wallis test with Dunn’s multiple comparisons test was used. *** *p* < 0.005; **** *p* < 0.001. (**b**) Fluorescence images of spheroid cross sections. Scale bar: 300 µm. DAPI: nuclear marker; Ki67: proliferation marker; CC3: apoptosis marker. (**c**) The percentage of Ki67-positive cells, which is indicative of the number of proliferative cells (N = 3). The data are presented as mean ± SD, and the Kruskal–Wallis test with Dunn’s multiple comparisons test was used. *** *p* < 0.005. (**d**) The percentage of CC3-positive cells, which is indicative of the number of apoptotic cells (N = 3). The data are presented as mean ± SD, and the Kruskal–Wallis test with Dunn’s multiple comparisons test was used. * *p* < 0.05.

As a more complex and representative model, cardiac organoids were generated and derived from induced pluripotent stem cells. Their viability and functional properties were monitored over time through size measurements, ATP levels and immunohistochemistry for the proliferation marker Ki67 and CC3 as marker for apoptosis, with samples collected at day 5, day 10 and day 20 after differentiation and reassociation. The first samples were collected on day 5 after seeding to allow the cells to form spheres that were compact enough to tolerate manipulation during sample collection (Figure 1a). In contrast to those of the cardiac spheroids, the 2D brightfield images of the cardiac organoids revealed no visual shrinkage of the spheres over time (Figure 1b). An average cardioid diameter of 556 ± 74 µm (mean ± SD) was measured on day 5 as the first timepoint. Over time, a significant increasing trend was detected with cardioid diameters ranging from 617 ± 71 µm (*p* < 0.05) to 710 ± 57 µm (*p* < 0.001) (mean ± SD) on days 10 and 20, respectively (Figure 1c). ATP level detection revealed a significant (*p* < 0.005) increase in the cardioid ATP level over time (Figure 1d). Their activity levels were confirmed by maintaining beating capacity over time (Figure 2a). Analysis of beating capacity revealed a significantly decreased contraction duration (*p* < 0.005), relaxation time (*p* < 0.01) and peak-to-peak time (*p* < 0.001 on day 10 and *p* < 0.005 on day 20), indicating that the organoids contracted faster over time and more frequently with a shorter relaxation time. However, when normalizing contraction duration and relaxation time data to the beat rate (peak-to-peak time), the observed significant differences were no longer detected (Figure S10a).

Additionally, the 10-to-10 transient parameter, indicative of the width of the peak top, increased significantly over time (*p* < 0.005 on day 10, *p* < 0.05 on day 20 and *p* < 0.001 compared with day 5), which means that the organoids were held longer in the contracted state. This trend remained present after normalization, although the difference between day 10 and day 20 during maturation appeared less pronounced (Figure S10a). Furthermore, immunofluorescence images of cardioid cross sections revealed an average of 21% Ki67-positive cells, which varied slightly over time, with a significant reduction on day 20 (15% positive cells (*p* < 0.005)) (Figure 2b,c and Figure S3). Moreover, an average maximum of 1% CC3-positive cells was measured over time, which was significantly lower on day 10 (*p* < 0.05, 0.7% positive cells) (Figure 2b,d and Figure S3), indicating that almost no apoptotic cells were present in the cardioid model than in the spheroid model (3% on day 3, 4% on day 7 and 5.5% on day 14) (Figure S2). Overall, these model validation results imply that the viability and functionality of our cardioid model are retained and that activity is improved over time during maturation, indicating that this model is better suited for evaluating preservation methods.

### 3.2. Effects of Cryopreservation on Cell Viability and Functionality

**Figure 3:**
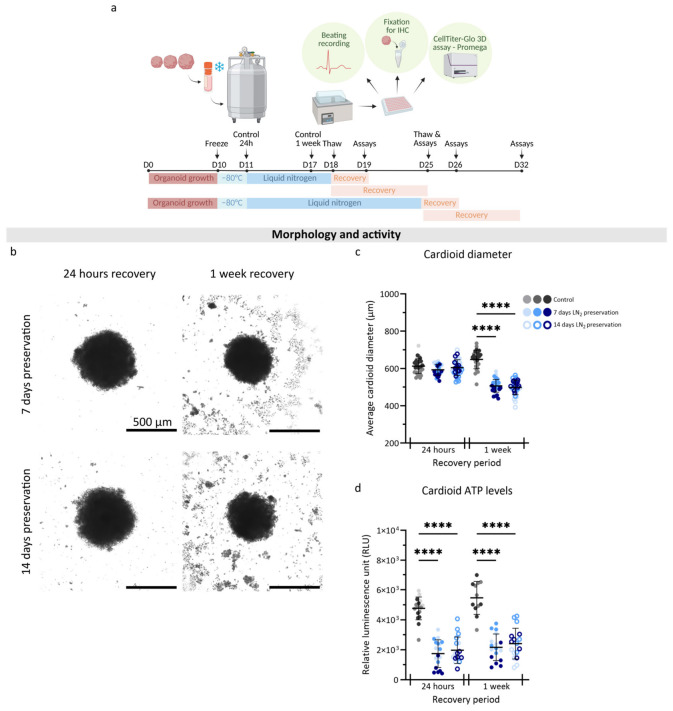
Cryopreservation of cardiac organoids. (**a**) Experimental overview of cardioid cryopreservation. D: day. IHC: immunohistochemistry. Created in BioRender. Bijnens, M. (2026) https://BioRender.com/tyoqxd8. (**b**) Brightfield images of cardiac cardioids during recovery upon preservation. Scale bar: 500 µm. (**c**) Averaged cardioid diameter over time after recovery. LN_2_: liquid nitrogen. Control: unpreserved cardioids. Shades of colors represent biological replicates (N = 2–3). The data are presented as the means ± SDs, and one-way ANOVA with Tukey’s multiple comparisons test was used. **** *p* < 0.001. (**d**) Averaged cardioid ATP levels over time during recovery. Shades represent biological replicates (N = 2–3). The data are presented as the means ± SDs. One-way ANOVA with Tukey’s multiple comparisons test was used. **** *p* < 0.001.

**Figure 4:**
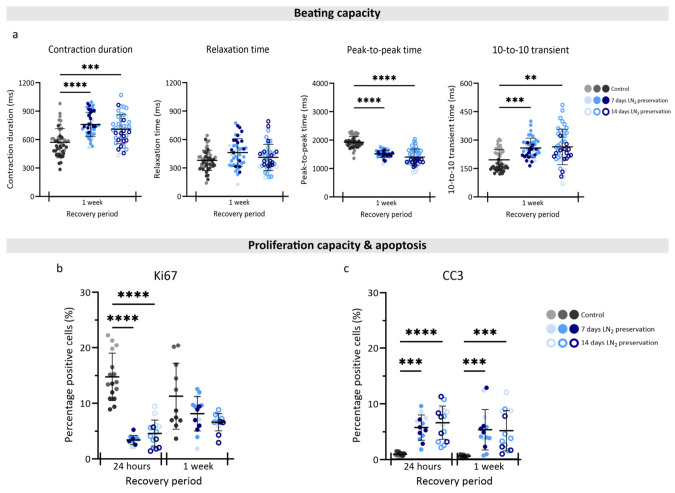
Beating, proliferation capacity and apoptosis of cardiac organoids upon cryopreservation. (**a**) Beating parameters of cardiac organoids upon preservation after one week of recovery: contraction duration, relaxation time, peak-to-peak time, 10-to-10 transient time in milliseconds (ms). Shades of colors represent biological replicates (N = 2–3). The data are presented as the means ± SDs. The Kruskal-Wallis test with Dunn’s multiple comparisons test was used. ** *p* < 0.01; *** *p* < 0.005; **** *p* < 0.001. (**b**) The percentage of Ki67-positive cells indicative for number of proliferative cells after preservation and 24 h or one week of recovery (N = 2–3). The data are presented as the means ± SDs, and the Kruskal-Wallis test with Dunn’s multiple comparisons test was used. **** *p* < 0.001. (**c**) The percentage of CC3-positive cells indicates the number of apoptotic cells after preservation and 24 h or one week recovery (N = 2–3). The data are presented as the means ± SDs, and the Kruskal-Wallis test with Dunn’s multiple comparisons test was used *** *p* < 0.005; **** *p* < 0.001.

Cardiac organoids were preserved in Cryostor^®^CS10 as a commonly used cryoprotectant for single iPSCs and tissues to evaluate the preservation effect on their viability and functionality based on previously described endpoints. Two different preservation incubation durations (7 days and 14 days) and two different recovery durations in a 37 °C humidified incubator (24 h and 1 week) were included to determine the optimal preservation protocol for our model (Figure 3a). Compared with those from the control group, organoid disintegration was visually observed, based on detached cell clusters surrounding the organoid in the well plate, and a decrease in size was measured via brightfield images taken from the cardioids preserved for 7 days and 14 days, which subsequently recovered for 1 week (Figure 3b). These visual observations revealed a significant decrease in the cardioid diameter after 1 week of recovery for both preservation periods (507 ± 50 µm (mean ± SD) after 7 days, *p* < 0.001; 498 ± 39 µm after 14 days, *p* < 0.001) compared with that of the controls (649 ± 50 µm) (mean ± SD) (Figure 3c). Furthermore, significantly lower ATP levels were detected after both 7 days (1745 ± 923 RLU after 24 h, 2165 ± 886 RLU after 1 week of recovery) (mean ± SD) and 14 days of preservation (1971 ± 877 RLU after 24 h, 2412 ± 1025 RLU after 1 week of recovery) (mean ± SD) than in unpreserved controls (4761 ± 761 RLU after 24 h, 5459 ± 1095 RLU after 1 week of recovery) (mean ± SD) (*p* < 0.001) (Figure 3d), indicating that the cells that were still present in the organoid after 24 h of recovery were not metabolically active. This finding was supported by the absence of beating capacity after 24 h of recovery (Figure 4a). Analysis of the beating capacity after longer recovery revealed a significantly increased contraction duration (*p* < 0.001 after 7 days and *p* < 0.005 after 14 days) and 10-to-10 transient time (*p* < 0.001 after 7 days and 14 days) and a significantly decreased peak-to-peak (*p* < 0.005 after 7 days and *p* < 0.01 after 14 days), indicative of longer contractions and increased contraction frequency that are held longer in the contracted state (Figure 4a). Normalization of beating parameters to peak-to-peak time revealed that alterations in beating behavior were in fact more pronounced than initially observed using the raw data (Figure S10b). Significant differences were detected for contraction duration, 10-to-10 transient time, and relaxation time after both one and three weeks of recovery (Figure S10b). In addition, normalization identified a significantly increased 10-to-10 transient time following fourteen days of cryopreservation and three weeks of recovery. These findings indicate that cryopreservation-induced alterations in beating behavior persist even after correction for differences in beat frequency.

We hypothesize that upon preservation, the nonmetabolically active cells in the organoid potentially underwent apoptosis with subsequent cell loss. This hypothesis is supported by immunohistochemistry, where a significantly greater number of apoptotic cells were present after 24 h, with 5.8% CC3-positive cells after 7 days of preservation (*p* < 0.005) and 6.6% CC3-positive cells after 14 days of preservation (*p* < 0.001), than the unpreserved controls (1% CC3-positive cells) (Figure 4c). This significant difference was still present after 1 week of recovery, with 5.4% CC3-positive cells remaining after 7 days of preservation and 5.2% CC3-positive cells remaining after 14 days of preservation compared with the controls (1% CC3-positive cells) (*p* < 0.005), indicating an insufficient recovery over time (Figure S4a,b). After 24 h recovery, significantly fewer proliferative cells were detected after 7 days of cryopreservation (3.4% Ki67-positive cells) and 14 days of cryopreservation (4.6% Ki67-positive cells) than in the unpreserved controls (14.7% Ki67-positive cells) (*p* < 0.001) (Figure 4b and Figure S4b), which indicates a reduced ability of the surviving cells to repopulate the organoid sample. Additionally, no significant differences were observed between 7 days and 14 days of cryopreservation, indicating that both cryopreservation periods induced adverse effects on cardioid viability and functionality.

### 3.3. Effects of Room Temperature-Based Preservation on Cell Viability and Functionality

**Figure 5:**
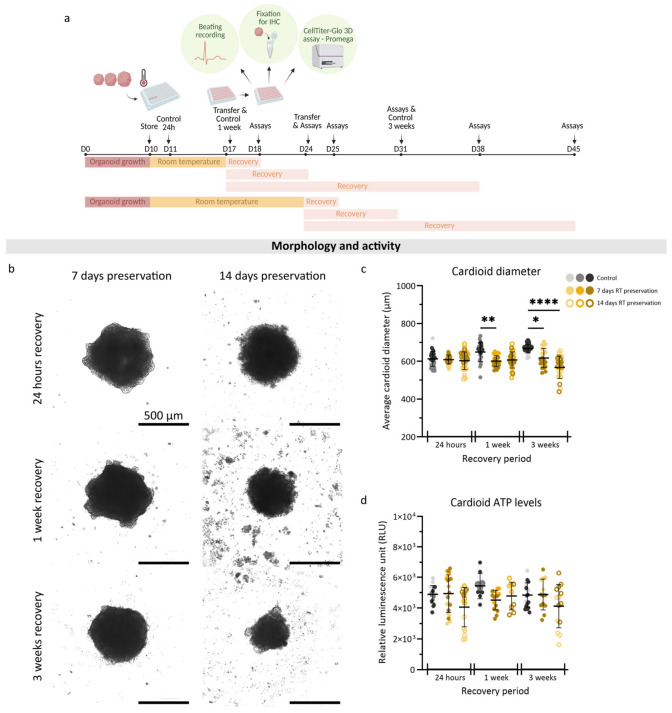
Room temperature-based preservation of cardiac organoids. (**a**) Experimental overview of cardioid preservation at room temperature. D: day. IHC: immunohistochemistry. Created in BioRender. Bijnens, M. (2026) https://BioRender.com/hj3srbm. (**b**) Brightfield images of cardiac cardioids during recovery upon preservation. Scale bar: 500 µm. (**c**) Averaged cardioid diameter over time after recovery. RT: room temperature. Control: unpreserved cardioids. Shades of colors represent biological replicates (N = 2–3). The data are presented as the means ± SDs, Brown-Forsythe and Welch ANOVA tests with Dunnett’s multiple comparisons test. * *p* < 0.05; ** *p* < 0.01; **** *p* < 0.001. (**d**) Averaged cardioid ATP levels over time during recovery. Shades represent biological replicates (N = 2–3). The data are presented as the means ± SDs. Brown-Forsythe and Welch ANOVA tests with Dunnett’s multiple comparisons test were used.

An alternative method for the standard cryopreservation of cells, a room-temperature-based preservation method was applied using the CellShip^®^ Cell Transport medium. This method was applied for 7 days and 14 days of preservation followed by various recovery periods (24 h, 1 week and 3 weeks) to investigate their viability and functionality based on the previously described endpoints (Figure 5a). The average cardioid diameter was significantly (*p* < 0.01) reduced after 7 days of preservation followed by 1 week of recovery (601 ± 29 µm) (mean ± SD) in a 37 °C humidified incubator compared with unpreserved controls (649 ± 50 µm) (Figure 5c). Compared with unpreserved organoids (667 ± 24 µm) (mean ± SD), those that recovered for 3 weeks had significantly lower average diameters after 7 days (617 ± 51 µm) (mean ± SD) (*p* < 0.05) and 14 days (568 ± 59 µm) (mean ± SD) (*p* < 0.001) of preservation (Figure 5c). However, when ATP levels were measured, no significant changes were detected during recovery despite the preservation periods (Figure 5d).

**Figure 6:**
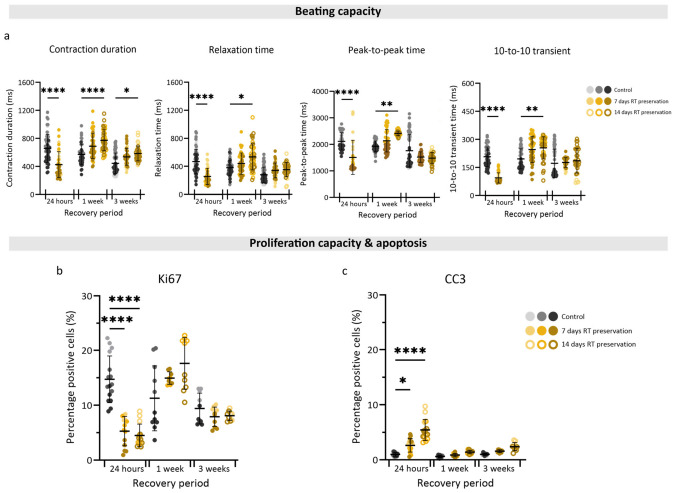
Beating, proliferation capacity and apoptosis upon room temperature preservation. (**a**) Beating parameters of cardiac organoids upon preservation after recovery: contraction duration, relaxation time, peak-to-peak time, and 10-to-10 transient time in milliseconds (ms). Shades of colors represent biological replicates (N = 2–3). The data are presented as the means ± SDs. The Kruskal-Wallis test with Dunn’s multiple comparisons test was used. * *p* < 0.05; ** *p* < 0.01; **** *p* < 0.001. (**b**) The percentage of Ki67-positive cells indicates the number of proliferative cells after preservation for 24 h, one week or three weeks (N = 2–3). The data are presented as the means ± SDs, and the Kruskal-Wallis test with Dunn’s multiple comparisons test was used. **** *p* < 0.001. (**c**) The percentage of CC3-positive cells indicates the number of apoptotic cells after preservation for 24 h, one week or three weeks (N = 2–3). The data are presented as the means ± SDs, and the Kruskal-Wallis test with Dunn’s multiple comparisons test was used. * *p* < 0.05; **** *p* < 0.001.

The beating capacity of the organoids preserved for 7 days at room temperature initially significantly decreased (*p* < 0.001) in terms of contraction duration, relaxation time, peak-to-peak time and 10-to-10 transient time, but these values progressively returned to levels comparable with those of the unpreserved controls during recovery (Figure 6a), suggesting successful recovery to conditions similar to those of the unpreserved controls after 1 week of recovery. Compared with those preserved for 7 days, the cardioids preserved for 14 days presented no measurable beating capacity, but this capacity was altered after 1 week of recovery (Figure 6a). At this recovery timepoint, all the included parameters, including contraction duration (*p* < 0.001), relaxation time (*p* < 0.05), peak-to-peak time (*p* < 0.01) and 10-to-10 transient time (*p* < 0.01), were significantly greater than those of unpreserved organoids (Figure 6a). The contraction duration remained significantly (*p* < 0.05) longer after 3 weeks of recovery compared to controls, but the other parameters were restored over time (Figure 6a). These beating results revealed that a shorter duration of preservation (7 days) resulted in a measurable beating capacity after 24 h of recovery, whereas recovery was delayed to 1 week when the mixture was preserved for 14 days at room temperature. Moreover, the cardioids that were preserved for 7 days recovered to a state similar to that of the unpreserved controls after 1 week, whereas the cardioids that were preserved for 14 days needed more time to recover. In contrast to cryopreservation date, normalization of the room temperature-based preservation data of individual parameters to peak-to-peak time showed that several initially observed differences after 24 h and one week of recovery, particularly for contraction duration and relaxation time, were no longer significant after correction for beat rate (Figure S10c). However, after three weeks of recovery, significant alterations in these parameters became apparent. A similar trend was observed for the 10-to-10 transient time, where the initially significant effect after one week recovery disappeared following normalization, while additional significant differences became detectable after three weeks recovery (Figure S10c). Importantly, we emphasize that the observed changes in individual beating parameters cannot be directly classified as beneficial or detrimental within the context of this model.

Immunohistochemistry results of the first recovery timepoint of 24 h showed an initial decrease (*p* < 0.001) in proliferation capacity upon 7 days of preservation (5.3% Ki67-positive cells) and 14 days of preservation (4.5% Ki67-positive cells) compared with the unpreserved controls (14.7% Ki67-positive cells), but it was restored over time during recovery (Figure 6b and Figure S7). Moreover, an average of 5.4% CC3-positive cells were detected after 24 h of recovery after 14 days, which was significantly greater (*p* < 0.001) than that of the unpreserved controls (1% CC3-positive cells). After a shorter preservation period of 7 days with a subsequent 24 h recovery, only 2.6% CC3-positive cells were detected (*p* < 0.05) (Figure 6c and Figure S7). The percentage of CC3-positive cells after 7 and 14 days of preservation restored with increasing recovery time.

Overall, these results indicate that in contrast to the standard cryopreservation methodology, the room temperature-based preservation method using CellShip^®^ Cell Transport medium was successful in retaining cardioid ATP levels and inducing less apoptosis after 1 week of recovery in a 37 °C humidified incubator after 7 days of preservation at room temperature.

## 4. Discussion

Baseline viability and functionality measurements during maturation revealed that our cardiac spheroid model is more sensitive to apoptosis than our cardiac organoids. This was indicated by the reduced cardioid size over time [55,56] and decreased generation of adenosine triphosphate (ATP) [55]. When comparing proliferation over time, cardiac organoids maintained their active and proliferative capacity better than cardiac spheroids did, possibly due to the self-renewal ability of the iPSC-derived organoid model [57,58,59]. This effect was confirmed by the increased cardioid diameter and enhanced ATP levels over time. Analysis of beating capacity revealed a significantly decreased contraction duration, relaxation time and peak-to-peak time, indicating that the organoids contracted faster over time and more frequently with shorter relaxation time. Additionally, the 10-to-10 transient parameter, indicative for the width of the peak top, increases significantly over time, which means that the organoids are held longer in the contracted state, possibly indicative of enhanced maturation [60,61,62,63]. However, when normalizing contraction duration and relaxation time data to the beat rate (peak-to-peak time), the observed significant differences were no longer detected (Figure S10a). Furthermore, immunofluorescence images of cardioid cross sections revealed Ki67-positive cells, which varied slightly over time, with a significant reduction on day 20 and an average maximum of 1% CC3-positive cells, indicating the almost absence of apoptotic cells in the cardioid model compared with the spheroid model. In contrast to the spheroids, the validation of the cardioid model revealed that the viability and functional properties of the organoids are retained and that their size, as well as their metabolic activity even improved over time during maturation, which implies their biological stability. Moreover, the application of the cardiac organoid model enables real-time monitoring of organoid functionality due to the beating capacity of the organoid as a first indication of successful recovery upon preservation. This highlights the high potential of using an organoid model while investigating preservation strategies.

Two different preservation strategies were tested on cardiac organoids, namely cryopreservation and room-temperature based preservation. The size of cryopreserved cardiac organoids decreased, at least partially through the loss of cells indicated by correlation analysis, which was observed visually only after longer recovery (Figure S9). However, it is important to note that analysis of 2D cross sections has inherent limitations when extrapolating to the entire 3D organoid structure. We hypothesize that additional time is needed for the preservation agent to be released outside the organoid while organoids that are already damaged prior to recovery require more time to be readapted to new environmental conditions [21]. Reduced ATP levels were detected, after both seven days and fourteen days of preservation, compared with those of the unpreserved controls, indicating that fewer cells were present or that the remaining cells in the organoid after 24 h of recovery were less active. We hypothesize that the nonactive cells in the organoid underwent apoptosis upon preservation with subsequent cell loss after 24 h of recovery, whereas some cells remained active or required more time to adapt to the environment. This finding was supported by the absence of beating capacity after 24 h of recovery, which was only detectable at 72 h (Figure S4a), and the initial reduction in the number of proliferative cells as well as the increased number of apoptotic cells upon preservation during recovery. The increased contraction duration and 10-to-10 transient time, also detectable when normalized to the beat rate (Figure S10b), are indicative of longer contracted stage. The significantly reduced peak-to-peak time is indicative of an increased contraction frequency. Based on these findings we hypothesize that the organoid needed to adapt to many changing factors after preservation, including new environmental conditions, and that it needed to compensate for stressors, with insufficient recovery observed over time. Importantly, we emphasize that the observed changes in individual parameters cannot be directly classified as beneficial or detrimental within the context of this model. The MUSCLEMOTION software provides a quantitative assessment of organoid movement as a mechanical response, allowing us to detect alterations in beating behavior without assigning functional superiority to specific parameter shifts. Evaluating viability or functional properties after a longer recovery of three weeks revealed no improvement in viability or functional properties based on previously described endpoints (Figures S5 and S10b). Additionally, no significant differences were observed between seven days and fourteen days of cryopreservation. When evaluating cardiac marker expression using immunohistochemistry on organoid cross sections upon cryopreservation, continued presence of cardiomyocytes was observed in cryopreserved cardioids after recovery (Figure S6). While normalized intensity analysis showed a transient significant decrease after seven days of cryopreservation followed by 24 h of recovery compared to controls, this difference was no longer observed after one week of recovery, suggesting restoration over time. Furthermore, no significant differences were detected between seven and fourteen days of cryopreservation. Although only one biological replicate was included due to technical and time limitations, these findings suggested that cryopreservation did not induce permanent alterations in cardiomyocyte presence within the organoids.

Overall, these findings indicate that both cryopreservation incubation periods adversely affect the viability and functional properties of cardiac organoids. However, we did not passage or replate the organoids upon cryopreservation during recovery, only the medium was changed, to evaluate whether the remaining cells could grow and repopulate the organoid. It appears that cryopreservation is suboptimal for cardiac organoids in our study design, likely due to limited cryoprotectant penetration and consequent cryodamage [20,27]. Interestingly, cryopreservation using highly permeable CPAs (DMSO and ethylene glycol) combined with nanoparticles and radiofrequency-based nanowarming has been applied to heart organoids in previous studies [21]. This synergistic strategy allowed rapid and uniform rewarming upon preservation, which was beneficial for overcoming the warming-rate limitation and thus maintaining cell viability. Alternatively, Becker et al. recently described an improved cryopreservation method for human iPSC-cardiomyocyte aggregates that relies on supplementation of preservation medium with 10% human serum albumin [64]. Although cell viability, contraction capacity and cardiac marker expression were maintained after five days of recovery, batch-to-batch variability was observed and the aggregates were very fragile immediately after thawing leading to cell loss due to handling, which affected their recovery rates. Overall, standardization of a relatively easy-to-implement cryopreservation strategy for organoids with retention of viability and function, while requiring a minimum recovery time, remains challenging. Currently, this is described as a common bottleneck for large-scale production, shipping and storage of various organoids [65].

Alternatively, a room temperature-based preservation method was evaluated. This method was previously validated on cell lines (single cells) for up to 120 h of preservation in cryovials with 2 mL preservation medium [34]. In our present study, we described for the first time the application of the Cellship^®^ Cell Transport medium on cardiac organoids for seven and fourteen days (without replenishment) when preserved in a small volume (200 µL) in 96-well plates as a novel preservation strategy. When the data were evaluated, brightfield images of the recovered organoids revealed minimal visually detectable morphological changes after seven days of preservation. However, a reduced organoid size was visually observed during recovery after fourteen days of preservation. When comparing the average diameter of preserved and unpreserved cardioids, unpreserved control organoids showed an increasing diameter over time. Compared with unpreserved organoids, preserved organoids that had recovered for one and three weeks presented significantly lower values, but the difference was not significant compared with the previous recovery timepoints. These findings indicate that the significant difference was only detectable due to increasing cardioid diameter over time in unpreserved controls, which is expected when cultured at 37 °C in a humidified incubator based on our baseline viability results.

Interestingly, no significant changes were detected over time during recovery when comparing cardioid ATP levels of preserved organoids with those of unpreserved controls, with limited biological variation between runs. Organoid activity upon preservation was further confirmed by their beating capacity, which was already measurable after 24 h of recovery in organoids preserved for seven days at room temperature. The beating profiles of the organoids that were preserved for seven days showed an initial significant decrease in contraction duration, relaxation time, 10-to-10 transient time and peak-to-peak transient time, but these values were restored over time to a comparable level to that of unpreserved controls. Moreover, these alterations were not detectable for the first two beating parameters, namely contraction duration and relaxation time, after 24 h and one week recovery when normalized to the beat rate (peak-to-peak time). However, alterations were observed upon three weeks recovery. This absence of changes in measured parameters upon seven days preservation to controls suggests successful recovery to a condition similar to that of the unpreserved controls after one week of recovery. Compared with those preserved for seven days, the cardioids preserved for fourteen days presented no measurable beating capacity after 24 h of recovery, but this capacity was restored after one week of recovery. At this recovery timepoint, all the included contraction parameters were significantly greater than those of unpreserved organoids, when not normalized, which may reflect a state of decreased workload or functional depression upon preservation due to transient functional adaptation during re-equilibration, but this is not specifically described in the literature during previous studies on cardiac organoids [11,66]. The contraction duration, as well as relaxation time and 10-to-10 transient time when normalized, remained significantly longer after three weeks of recovery than that of the controls, suggesting nearly complete recovery to maintain activity [11,67,68]. Overall, these beating results showed that preservation for seven days resulted in measurable beating capacity after 24 h of recovery, whereas this was delayed to one week of recovery after preservation for fourteen days at room temperature. Moreover, the cardioids that were preserved for seven days recovered to a state similar to that of the unpreserved controls after one week. This distinction underlies our interpretation that, within the context of our model and experimental setup, a seven-day room temperature preservation period better maintains baseline beating behavior after 24 h recovery compared to a longer (fourteen-day) preservation period. However, we emphasize that the observed changes in individual parameters cannot directly be classified as beneficial or detrimental for this model. The MUSCLEMOTION software tool offers a quantitative assessment of organoid movement as a mechanical response, which allows the detection of beating behavior alterations without assessing functional superiority based on specific parameter shifts when evaluating the output. Our interpretation is based on the overall deviation from unpreserved control behavior. The alterations observed after cryopreservation indicate a shift in beating dynamics relative to unpreserved controls, whereas the absence of such changes following seven days of room temperature preservation suggests a closer resemblance to baseline conditions. We therefore interpret this as indicative of a more preserved beating phenotype, rather than assigning functional superiority to specific parameter values.

We hypothesize that this was the result of a reduced metabolic state induced by the room temperature-based preservation method, in which the cells in the organoid are less active, producing less ATP and having minimal proliferation capacity. Recovery from this prolonged reduced metabolic state after fourteen days could be more difficult and thus require more time. This hypothesis is further supported by the absence of measurable beating capacity after 24 h of recovery after fourteen days of preservation and by the initial increase in the number of apoptotic cells and decrease in the number of proliferative cells. Interestingly, these deviations were restored after one week of recovery, at which point more cells were actively proliferating, with almost no apoptotic cells present in the organoid that had a beating capacity similar to that of the unpreserved controls. Additionally, cardiac marker expression, evaluated through immunohistochemistry, was maintained upon room temperature-based preservation and recovery (Figure S8). This indicates that applying this preservation strategy did not induce permanent alterations in the predominant cardiomyocyte population of the cardiac organoids. However, beyond the readouts included in this study, future work could benefit from integrating flow cytometry and/or single-cell RNA sequencing to evaluate the effect of this preservation approach to the multicellular composition. Furthermore, metabolic profiling approaches can be integrated in the future to assess mitochondrial respiration and glycolytic activity to verify our hypothesis regarding the reduced metabolic state upon preservation. Furthermore, cell-based assays specifically targeting the detection of reactive oxygen species (ROS) would give more insights in the metabolic effects occurring when applying this preservation strategy.

Overall, these results indicate that the preservation strategy using CellShip^®^ Cell Transport medium was successful in retaining cardioid viability, based on stable ATP levels and less apoptosis, as well as retainment of beating capacity, starting from 24 h to one week of recovery in a 37 °C humidified incubator after seven days of preservation at room temperature. Interestingly, another commercially available compound, HypoThermosol (STEMCELL), was recently applied for three days on cardiac tissue constructs consisting of human iPSC-derived cardiomyocytes and human cardiac fibroblasts embedded in a hydrogel [5]. Compared with cryopreservation, hypothermic preservation (2–8 °C) using HypoThermosol showed good results in terms of the maintenance of cell viability and functionality, indicating the potential of this preservation solution for short-term storage and/or transport, yet it was applied for a limited period of three days. Overall, this type of hypothermic solution is promising for use with 3D cellular models, but the need for reduced environmental temperature (2–8 °C) while preserving still highlights the remaining lack of solutions that can be used at room temperature (20–25 °C) and/or can be applied for long-term preservation while maintaining contractility and cell viability [5]. Our recent findings on the room temperature-based preservation of cardiac organoids using Cellship^®^ Cell Transport medium for seven days serve as a first step toward overcoming problem and contribute to the research gap in room temperature preservation for 3D cardiac models.

This room temperature-based preservation strategy for 3D cellular models could induce emerging innovations when compatible with high-throughput screening and biobanking, which would allow researchers to make off-the-shelf 3D cellular models that can be used on demand. This could solve technical challenges in experimental set-ups, for example, in drug screenings where many models are needed at the same time [1,69,70,71,72]. When this room temperature-based preservation method is scaled to the tissue and/or organ level, this could provide a solution to the global shortage of organ availability for organ transplantation. When a match is found between a donor and a recipient, suitable donated organs are not delivered on time because of the large distances between the donor and the recipient. With a transport window of only four to six hours, many potential suited grafts become unusable and are discarded, which leads to a growing list of patients waiting for their new organ(s) [73]. As a standard method for organ preservation, static cold ischemic preservation upon flush-out with preservation solution or the innovative method of supercooling still requires the temperature to be lowered to a certain range of approximately zero degrees and does not provide adequate protection to the donated organ during prolonged cold storage [74,75,76]. Since fewer organs become available and organ transport is often time-consuming, improving organ survival during the transportation of human organs is necessary. Room temperature-based preservation could overcome these technical difficulties related to the preservation of organs at cold temperatures and could increase the time needed for organ transport. Nevertheless, since we only tested this preservation method on iPSC-derived cardiac organoids, the application of this method to a wide range of different 3D models of different cell types representing various organs remains to be investigated. Applying this preservation strategy to larger tissues and organs would require technical adaptations related to volumes of preservation media and its supplementation specifically for human organs, as well as challenges related to sterile storage. Moreover, the complex architecture of human organs including different cells and compartments, presents some extra challenges to preservation strategies. Beyond cardiac organoids, the application of preservation strategies to other advanced tissue models, including organ slices, represents an important future perspective. Organ slices are increasingly used in translational and pharmacological research due to their ability to retain native tissue architecture and multicellular complexity [37,38,39,40,41]. In addition, preservation approaches may also be highly relevant for diseased organoids and pathological tissue models [37,40,42,43]. However, diseased tissues often exhibit altered cellular composition, extracellular matrix organization, metabolic activity, oxygen consumption, and stress sensitivity compared to healthy tissues, which may influence preservation efficacy and recovery capacity [44,45,46,47]. Therefore, preservation methods optimized for healthy organoids may not be directly transferable to more complex and sensitive tissue systems. Extensive optimization and validation of preservation conditions, including preservation medium composition, oxygen and nutrient diffusion, recovery protocols, tissue thickness, and preservation duration, will be required for each specific tissue type and disease context. Furthermore, additional functional and metabolic characterization will be necessary to confirm maintenance of tissue integrity and disease-specific phenotypes following preservation and recovery.

In addition to Earth applications, 3D cellular models derived from human cells can be used to mimic adverse health effects occurring in the bodies of astronauts in space. Investigating the harmful effects of space travel on the human body, especially on specific organs including the heart, is crucial for performing health risk assessments of astronauts during space missions [77,78,79,80,81]. However, keeping cultures viable while travelling to the international space station (ISS) or during deep space missions remains challenging and often costs substantial resources during mission planning and execution. Creating a technology that allows preservation of 3D cellular models while retaining cell viability and functionality would therefore solve technical barriers and lead to innovation in biological space research during future long-duration space missions. Based on the selective readouts used in this study, our room temperature-based preservation strategy retained the viability and functional properties of a 3D cardiac model after seven days of preservation. This time window of seven days would cover the required time for payload preparation when sending experiments on a space mission, for example, to the ISS to investigate ageing-related cardiovascular effects in space. The methodology of room temperature-based preservation would therefore simplify preservation conditions and reduce the payload weight and financial cost. As an emerging technology, organ-on-chips (OoCs) are advanced 3D microfluidic platforms that hold great promise as tools for application in tissue engineering and regenerative medicine on Earth as well as in space research [82,83]. Preserving these OoCs at room temperature via microfluidic channels would allow gradual delivery and penetration of preservation medium in a complex 3D system for optimal and easy-to-implement preservation and extended usage time of the model. The use of in vitro conditions, especially prolonged microgravity and high-radiation environments, may not fully represent the complex architecture of human organs in space. Therefore, scaling up the preservation approach for larger tissues or integrated organ systems and evaluating long-term stability under space radiation exposure are critical future steps.

Limitations of the current study include the exclusive focus on cardiac organoids, by using a single hiPSC line. As such, the generalizability of our results remains to be established. We consider this work as a preliminary step, and further studies will be necessary to assess the applicability of this approach across additional hiPSC lines and a broader range of microtissue models. While our results suggest potential for ambient temperature-based preservation of 3D cell constructs, additional validation is required before broader conclusions can be drawn regarding its utility. Future investigations will be essential to determine whether this strategy can reliably complement existing preservation methods and offer practical advantages in specific experimental contexts. As one of the important readouts, viability is described in this study on the basis of ATP measurements via the CellTiter-Glo^®^ 3D Cell Viability Assay. In addition to the presence of proliferative (Ki67-positive) cells and the number of apoptotic (CC3-positive) cells, these findings provided an indication of cell viability in our cardiac organoid model. Furthermore, cell functionality was evaluated based on their beating capacity. However, additional readouts, such as cell counting and live/dead assays, metabolic assays, necrotic core evaluation, flow cytometry and gene expression analysis, could be implemented to confirm the results and hypotheses on cell viability, cell loss and cell death.

Another limitation of the current study is the inclusion of a relatively short preservation periods compared with the long-term preservation of months to years of cryopreservation. Cryopreservation is the standard approach for long-term cell storage. However, we intentionally aligned the storage durations for both room temperature and liquid nitrogen conditions to enable a direct comparison of short-term storage performance. While room temperature-based preservation using CellShip^®^ has previously been validated for suspended cells up to 120 h, our study extends this approach to a more complex 3D cell model for 7 and 14 days. This demonstrates the potential applicability of the method over longer periods and in more advanced biological systems. However, this should be first tested in the future by adapting the experimental setup. Furthermore, we want to highlight the importance of scaling and container-related parameters in the context of ambient temperature preservation and logistics, since factors such as container geometry, volume-to-tissue ratio and gas exchange may influence preservation outcomes. In the present study, room temperature preservation was carried out using 96-well plates that were air-tightly sealed and placed in a transport box to minimize external disturbances, such as vibrations from routine laboratory activity. This setup was chosen to maintain stable conditions throughout the preservation period while preventing medium evaporation. Furthermore, it enables a simple and low-effort workflow, requiring minimal handling and relatively small volumes of preservation medium. The use of 96-well plates is consistent with the standard culture format of our organoids, as they are generated, matured, and maintained in the same type of plate, thereby minimizing reducing potential sources of variability. We acknowledge that this simplified setup does not account for potential effects of medium exchange during storage, nor does it systematically evaluate variables relevant to scaling, such as container type, volume, or oxygen availability. Adjustments to these parameters, for example, periodic medium replacement or the use of alternative container formats, may influence (long-term) preservation outcomes. However, a systematic investigation of these factors is beyond the scope of the present study. The current work should therefore be regarded as a proof-of-concept, demonstrating that room temperature preservation in a simple, static microplate-based format can support the recovery of organoid viability and function. Further studies will be necessary to evaluate the robustness of this approach under varying storage conditions and to determine its scalability for broader applications.

Overall, this innovative preservation method for 3D cardiac organoids at room temperature holds great promise for various types of human organoids and, in the future, it could also be applied to human tissues and organs after excessive optimization and validation for translation. Future research is therefore needed to evaluate the preservation effects on other cell types and more complex cellular models, including tissues, organ slices and organs, to optimize preservation conditions and prolong preservation time. In this way, a common standard preservation methodology can be generated that finds its application in tissue engineering, regenerative medicine and biomedical research on Earth and in space.

## 5. Conclusions

In this study, we demonstrate that the sustained beating capacity, low apoptotic burden, and increased metabolic activity of cardiac organoids during maturation indicate their suitability for evaluating preservation strategies and enabling real-time, noninvasive monitoring of recovery following preservation.

Both seven- and fourteen-day conventional cryopreservation periods resulted in reductions in viability and functional performance, characterized by size reduction, decreased ATP levels, delayed recovery of beating capacity, and increased apoptosis. In contrast, room temperature-based preservation via CellShip^®^ Cell Transport medium effectively maintained cardiac organoid ATP levels and beating properties, with less apoptosis, particularly after recovery after seven days of preservation.

Overall, these findings indicate that room temperature-based preservation is a promising alternative preservation strategy to cryopreservation for the short-term storage of 3D cardiac organoids, offering retention of viability and functional properties, while avoiding freezing and thawing-induced cellular damage. This approach holds great potential for applications requiring scalable, high-throughput preservation with less specialized infrastructure, including biobanking, drug screening, and distributed experimental workflows. While the present study is limited to iPSC-derived cardiac organoids, the results provide a proof-of-concept for extending this preservation strategy to other organoid systems, organ-on-chip platforms, and, with further optimization, organ slices and larger tissues. Future studies will be needed to evaluate the applicability of this method across diverse tissue types, to optimize and prolong preservation durations, and to assess its scalability toward clinically relevant tissue and organ preservation.

## Data Availability

The data supporting this study are available within the article and its Supplementary Materials. Further inquiries can be directed at the corresponding author. The data that support the findings of this study are not openly available for reasons of sensitivity and are available from the corresponding author upon reasonable request. Data are located in controlled access data storage at the Belgian Nuclear Research Center (SCK CEN).

## Supplementary Materials

The following supporting information can be downloaded at https://www.mdpi.com/article/10.3390/cells15121065/s1. Figure S1: Cardiac spheroid validation by baseline viability and functionality measurements; Figure S2: Spheroid validation over time during maturation including Staurosporine-treated control. Figure S3: Cardioid validation over time during maturation including Staurosporine-treated control. Figure S4: Cryopreservation of cardiac organoids. Figure S5: Cryopreservation of cardiac organoids with three weeks recovery. Figure S6: Presence of cardiomyocytes in cryopreserved cardioids. Figure S7: Proliferation capacity and apoptosis of cardioids preserved at room temperature. Figure S8: Presence of cardiomyocytes in cardioids preserved at room temperature. Figure S9: Correlation analysis between cardioid size and nuclei count. Figure S10: Normalization of beating parameters.
