# Supplementary material for: Evaluation of a Room-Temperature Preservation Method Maintaining Viability and Function in Human Cardiac Organoids
Source: Cells. 2026 Jun 11;15(12):1065. doi: 10.3390/cells15121065 (PMC13297580; doi:10.3390/cells15121065)
Supplement: Supplementary file 1 [file cells-15-01065-s001.zip › cells-4325491-supplementary.pdf]

Supplementary materials

2. Materials and methods

2.1 Cell culture

2.1.1. Spheroids

Cardiac spheroids were generated from immortalized cell types, including human cardiomyocytes (AC16, Sigma-Aldrich, SCC109), human cardiac fibroblasts (IM-HCF, Innoprot, P10453-IM) and human telomerase-immortalized endothelial cells (HCAEC or TICAE, gifted by prof. dr. Kenneth Raj from Public Health England) at a ratio of 70% AC16, 20% IM-HCF, 10% HCAEC. Cells were cultured in complete MesoEndo growth medium (Cell Applications, INC., 212-500) and incubated at 37 °C in a humidified atmosphere containing 95% air and 5% CO_2 (Greiner Bio-one) incubator. The Moxi Cell Counter (Corning Incorporated Life sciences) was applied for cell counting, and cells were passaged at 80-90% confluence using 1X 0.05% Trypsin-EDTA (GIBCO, 25300-062). Cardiac spheroids were grown for three, seven and fourteen days in agarose molds generated from micro-molds (MicroTissues® 3D Petri Dish®, Merck) by seeding the cell mixture at a recommended seeding density of 35 000 cells per spheroid. Spheroids were maintained in MesoEndo growth medium (Cell Applications, INC., 212-500). Spheroid growth was followed through size measurements over time based on brightfield images taken with the Leica Application Suite microscope software platform (Leica Microsystems, Wetzlar, Germany).

2.2 Preservation method

Cryopreservation and thawing

Ten-days old cardiac organoids were resuspended in cryopreservation medium including Cryostor®CS10 (STEMCELLTM Technologies, 100-1061). Standard cryopreservation following a slow freezing protocol was initiated by transferring the 3D cardiac constructs into a cryovial (Cryo.S, 2 mL, round bottom, Greiner Bio-one) with 1 mL cryopreservation medium, with subsequent cooling and storage overnight in a CoolCell™ freezing vial container at -80 °C to ensure a slow cooling rate of -1 °C min-1. After overnight incubation, the cryovials were transferred to a liquid nitrogen freezer (-196 °C), where they were stored for an additional seven or fourteen days. Post-preservation, samples were thawed in a 37 °C water bath until only a small ice clump remained after which cardiac constructs were transferred to a Corning® 96-well Black/Clear Round Bottom Ultra-Low Attachment Surface Spheroid Microplate (Corning, 4520) upon washing with pre-warmed maintenance medium supplemented with 10 μM ROCK inhibitor Y-27632 (STEMCELL, 72304). After 24 hours, medium was changed to standard

maintenance medium. One day (24h) and one week (1 week) post thawing, viability and functionality measurements were performed.

3. Results

3.1 3D cardiac model validation

An average spheroid diameter of 630 ± 55 µm was measured at day 3 as first time point. However, as already observed through brightfield microscopy pictures (Figure S1b), a decreasing trend was measured over time with spheroid diameters of 564 ± 71 µm to 470 ± 46 µm on day 7 and day 14 respectively (Figure S1c). This significant ($p<0.001$) reduction in spheroid diameter is potentially indicative for cell death and/or subsequent loss of cells due to compactness. When evaluating adenosine triphosphate (ATP) levels over time using the CellTiter-Glo® 3D assay, a similar decreasing trend is observed over time with a 42% significant ($p<0.001$) reduction of luminescence signal at day 14 (1272 ± 547 RLU) compared to day 3 (2211 ± 726 RLU) and 34% reduction compared to day 7 (1942 ± 447 RLU). Immunohistochemistry for the proliferation marker Ki67 was applied on spheroid cross sections to investigate the proliferation capacity of the cardiac spheroids during their maturation over time. The results showed a significant decrease of percentage Ki67-positive cells after 7 days ($p<0.05$) and 14 days ($p<0.005$) compared to day 3 (Figures S2a, b). However, immunofluorescence images showed more Ki67-positive cells at the periphery compared to the center of the sphere (Figures S2a, b) indicating that the cells at the most outer layer of the sphere are more proliferative than the cells in the center. Cleaved caspase-3 (CC3), as marker for apoptosis, was used to verify cell death and to validate cell viability during spheroid maturation. Results showed a significant ($p<0.005$) increase in CC3-positive cells over time at day 14 compared to day 3 (Figures S2a, c), indicating that the cells in the spheroids became apoptotic over time and thus generate lower ATP levels. Overall, these results suggest that the viability of our cardiac spheroids diminishes over time during maturation and therefore most likely cannot withstand the stresses of the preservation methodology, due to low ATP production and apoptotic cells already present after one week of culture.

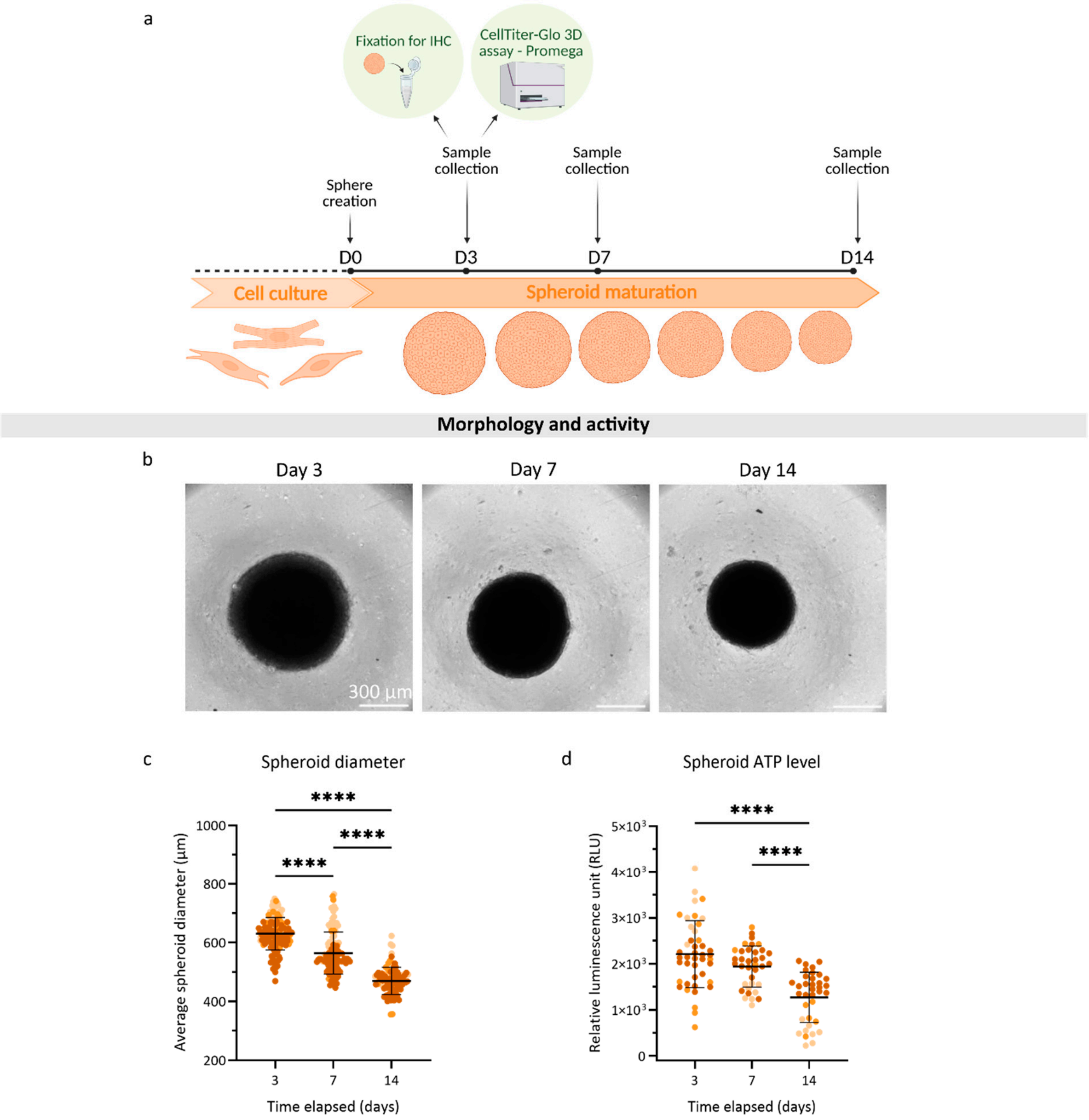

Figure S1: Cardiac spheroid validation by baseline viability and functionality measurements (**a**) Overview of spheroid baseline experiment. D: day. IHC: immunohistochemistry. Created in BioRender. Bijnens, M. (2026) https://BioRender.com/a0zp5cp. (**b**) Brightfield pictures of cardiac spheroids over time during maturation. Scale bar: 300 µm. (**c**) Averaged spheroid diameter over time during maturation. Shades represent biological replicates (N=3). The data are presented as the means ± SD, and the Kruskal-Wallis test with Dunn's multiple comparisons test was used. ****p<0.001. (**d**) Averaged spheroid ATP levels over time during maturation. ATP: adenosine triphosphate. Shades represent biological

replicates (N=3). The data are presented as the means ± SDs. One-way ANOVA with Tukey's multiple comparisons test was used. ****p<0.001.

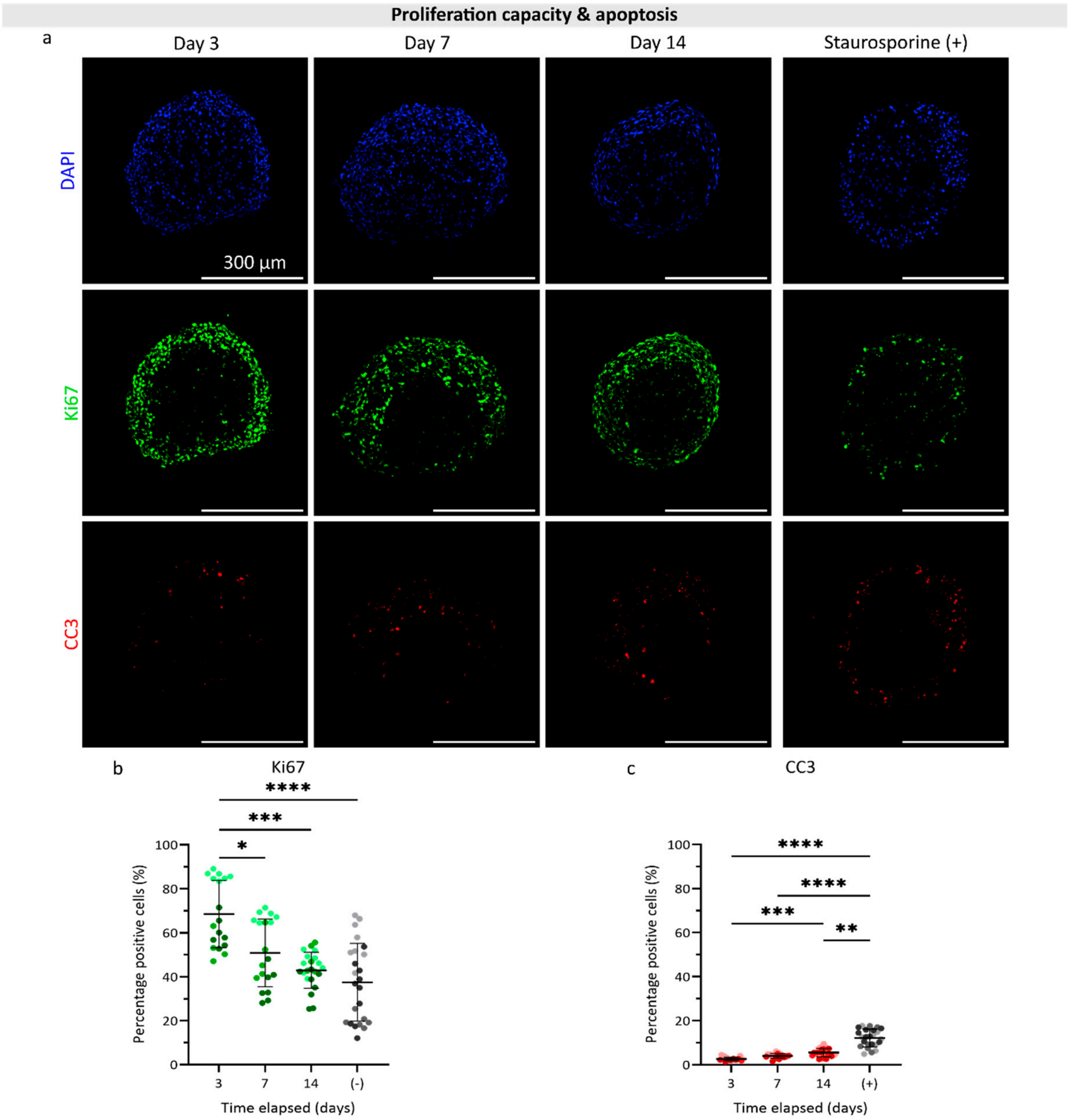

Figure S2: Spheroid validation over time during maturation including Staurosporine-treated control. (**a**) Immunofluorescence images of spheroid cross sections. Scale bar: 300 µm. DAPI: nuclear marker; Ki67: proliferation marker; CC3: apoptosis marker. Staurosporine (+) is a positive control for apoptosis and an on-proliferative/apoptotic control to demonstrate loss of Ki-67 expression. (**b**) The percentage of Ki67-positive cells, which is indicative of the number of proliferative cells (N=3). (-): Staurosporine-treated spheroid as non-proliferative/apoptotic control to demonstrate loss of Ki67

expression. The data are presented as the means ± SD, and the Kruskal-Wallis test with Dunn's multiple comparisons test was used. *p<0.05; ***p<0.005; ****p<0.001. (**c**) The percentage of CC3-positive cells, which is indicative of the number of apoptotic cells (N=3). (+): Staurosporine-treated spheroid positive control. The data are presented as the means ± SD, and the Kruskal-Wallis test with Dunn's multiple comparisons test was used. **p<0.01; ***p<0.005; ****p<0.001.

3.2 Cardioid baseline viability

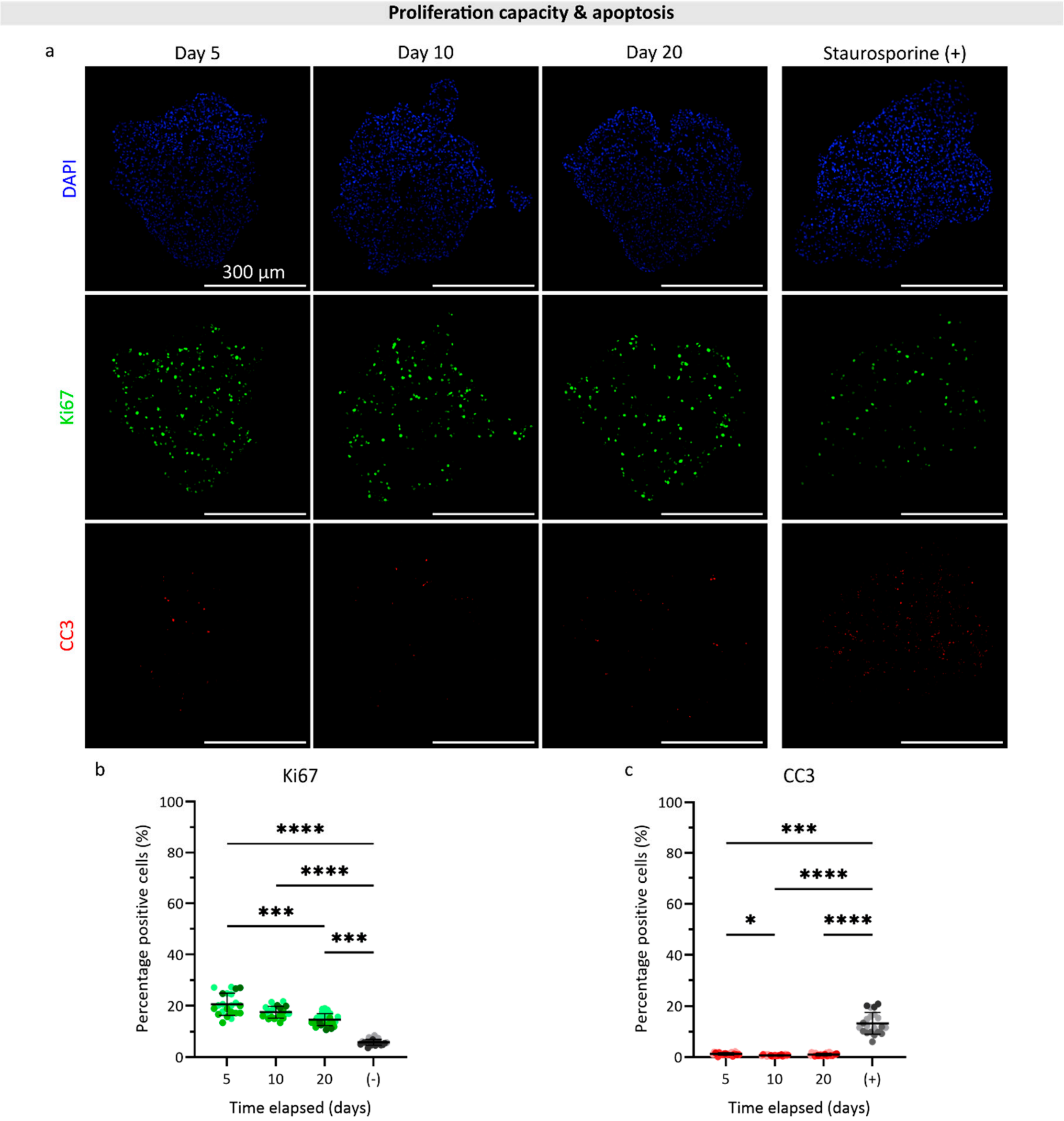

Figure S3: Cardioid validation over time during maturation including Staurosporine-treated control. (**a**) Immunofluorescence images of cardioid cross sections. Scale bar: 300 µm. DAPI: nuclear marker; Ki67: proliferation marker; CC3: apoptosis marker.

Staurosporine (+) as positive control for apoptosis and as non-proliferative/apoptotic control to demonstrate loss of Ki67 expression (-). (**b**) The percentage of Ki67-positive cells, which is indicative of the number of proliferative cells (N=3). (-): Staurosporine-treated cardioid as non-proliferative/apoptotic control to demonstrate loss of Ki67 expression. The data are presented as the means ± SD, and the Kruskal-Wallis test with Dunn's multiple comparisons test was used. ***p<0.005; ****p<0.001. (**c**) The percentage of CC3-positive cells, which is indicative of the number of apoptotic cells (N=3). (+): Staurosporine-treated cardioid positive control. The data are presented as the means ± SD, and the Kruskal-Wallis test with Dunn's multiple comparisons test was used. *p<0.05; ***p<0.005; ****p<0.001.

3.3 Cryopreservation

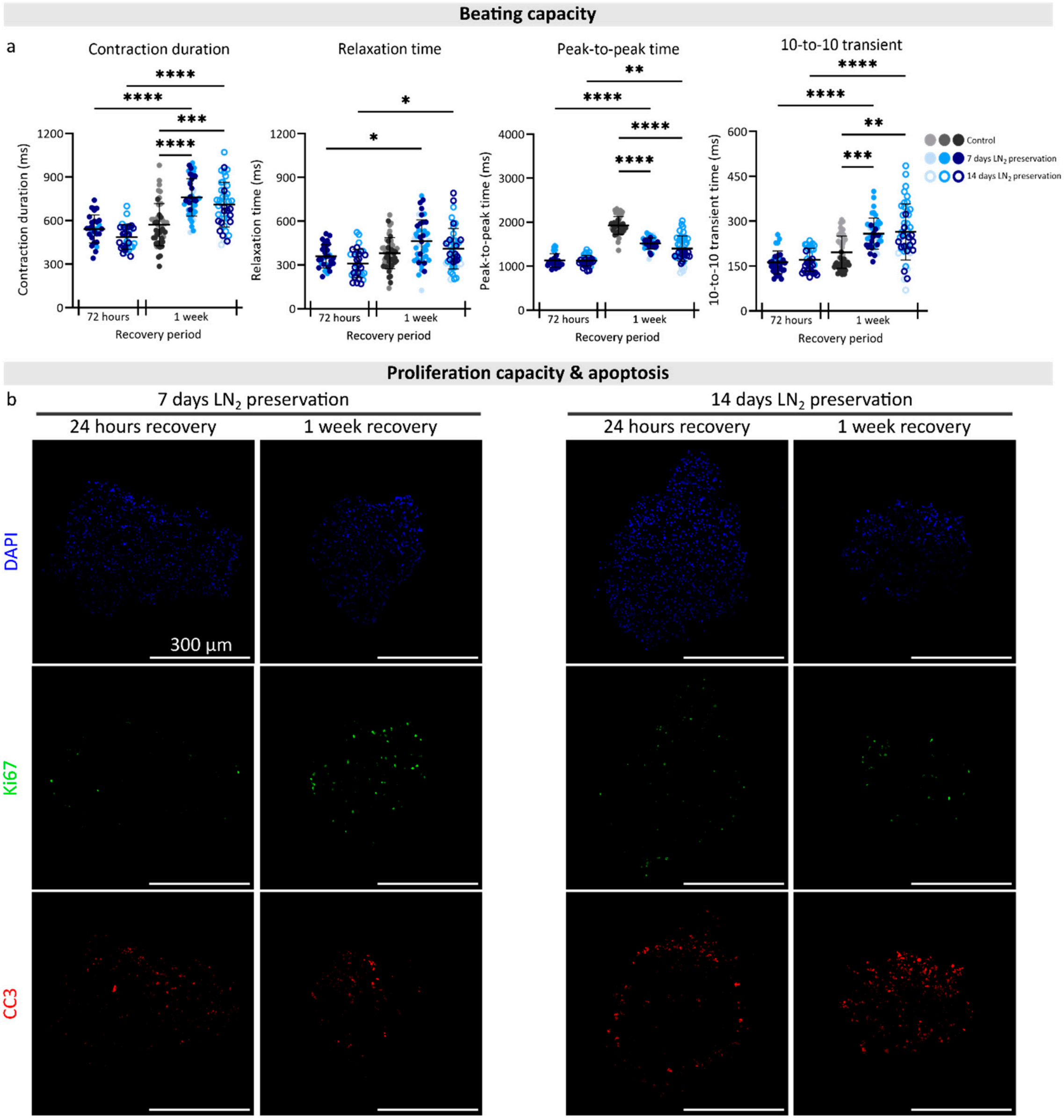

Figure S4: Cryopreservation of cardiac organoids. (**a**) Beating parameters of cardiac organoids after cryopreservation in liquid nitrogen (LN_2) after 72 hours and one week recovery: contraction duration, relaxation time, peak-to-peak time, 10-to-10 transient time in milliseconds (ms). After 72 hours of recovery, beating was measurable. Shades represent biological replicates (N=2-3). The data are presented as the means ± SD, and the Kruskal-Wallis test with Dunn's multiple comparisons test was used. *p<0.05; **p<0.01; ***p<0.005; ****p<0.001. (**b**) Immunofluorescence images of cardioid cross sections upon cryopreservation in LN_2 after 24 hours and one week recovery. Scale bar: 300 µm. DAPI: nuclear marker; Ki67: proliferation marker; CC3: apoptosis marker.

3.3.1 Extended recovery time of three weeks for cryopreserved cardioids

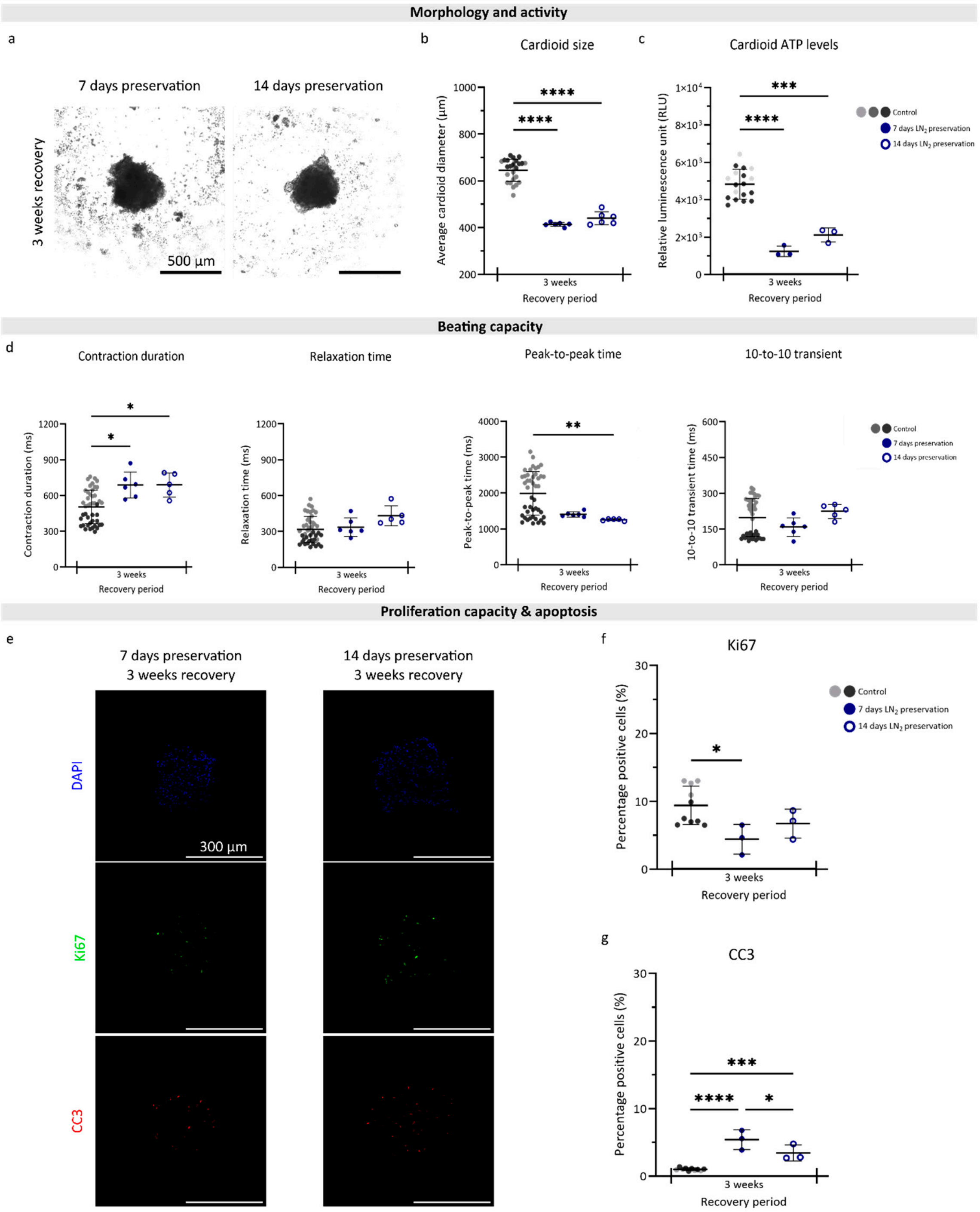

Figure S5: Cryopreservation of cardiac organoids with three weeks recovery. (**a**) Brightfield pictures of cardiac organoids preserved for 7 days and 14 days in liquid nitrogen (LN_2) and recovered for three weeks. Scale bar: 500 µm. (**b**) Averaged cardioid diameter after cryopreservation and three weeks recovery. (**c**) Averaged cardioid ATP levels after cryopreservation and three weeks recovery. The data are

presented as the means ± SD, and the one-way ANOVA with Tukey's multiple comparisons test was used. *p<0.05; ***p<0.005; ****p<0.001. (**d**) Beating parameters of cardiac organoids after cryopreservation in liquid nitrogen (LN_2) after three weeks recovery: contraction duration, relaxation time, peak-to-peak time, 10-to-10 transient time in milliseconds (ms). The data are presented as the means ± SD, and the Kruskal-Wallis test with Dunn's multiple comparisons test was used. *p<0.05; **p<0.01; ***p<0.005; ****p<0.001. (**e**) Immunofluorescence images of cardioid cross sections upon cryopreservation in LN_2 after three weeks recovery. Scale bar: 300 μm. Shades represent biological replicates (N=1). DAPI: nuclear marker; Ki67: proliferation marker; CC3: apoptosis marker. (**f**) The percentage of Ki67-positive cells, which is indicative of the number of proliferative cells (N=1). (**g**) The percentage of CC3-positive cells, which is indicative of the number of apoptotic cells (N=1). The data are presented as the means ± SD, and the one-way ANOVA with Tukey's multiple comparisons test was used. *p<0.05; ***p<0.005; ****p<0.001.

3.3.2 Immunohistochemistry for cardiomyocyte-specific marker: cardiac troponin T

We performed an additional immunostaining for cardiac troponin T to specifically evaluate cardiomyocytes, the predominant cell type in the organoids (approximately 80% of the total cell population), and to provide cell type–specific insight.

Supplementary figure S6 shows the presence of cardiomyocytes in cryopreserved cardioids upon recovery. Normalized intensity data showed a significant decreased signal upon 7 days cryopreservation and 24 hours recovery compared to control samples. However, this difference was restored after one week recovery. Of note, due to time limitations and technical restrictions, only one biological replicate was included. No significant differences were observed between 7 days cryopreservation compared to 14 days preservation. Overall, this data indicates no permanent alterations of cardiomyocyte presence, which is the most prominent cell type in the organoid, upon cryopreservation.

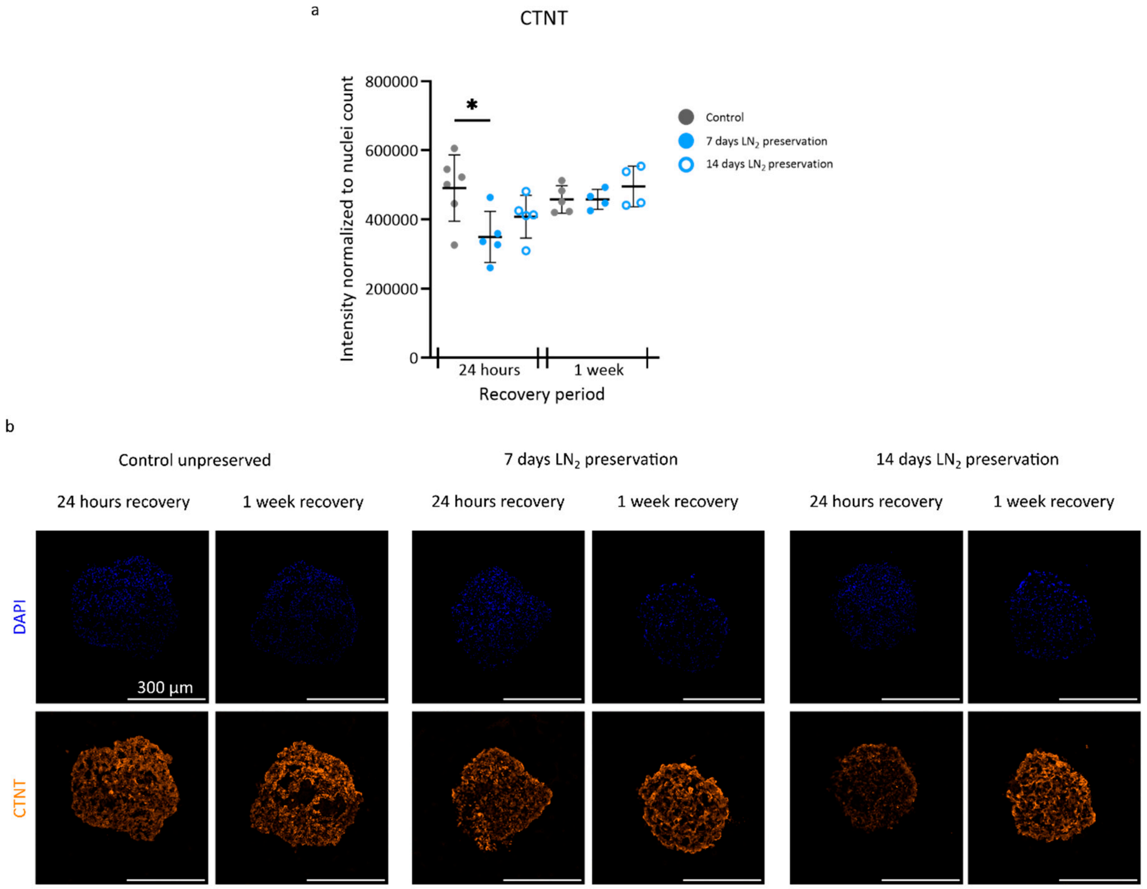

Figure S6: Presence of cardiomyocytes in cryopreserved cardioids. (**a**) The intensity of cardiac troponin T-positive cells, which is indicative of cardiomyocytes (N=1). The data are presented as the means ± SD, and the one-way ANOVA with Tukey's multiple comparisons test was used. *$p<0.05$. (**b**) Immunofluorescence images of cardioid cross sections upon cryopreservation after 24 hours and one week of recovery. Scale bar: 300 µm. DAPI: nuclear marker; CTNT: cardiac troponin T, cardiomyocyte-specific marker.

3.4. Room temperature-based preservation

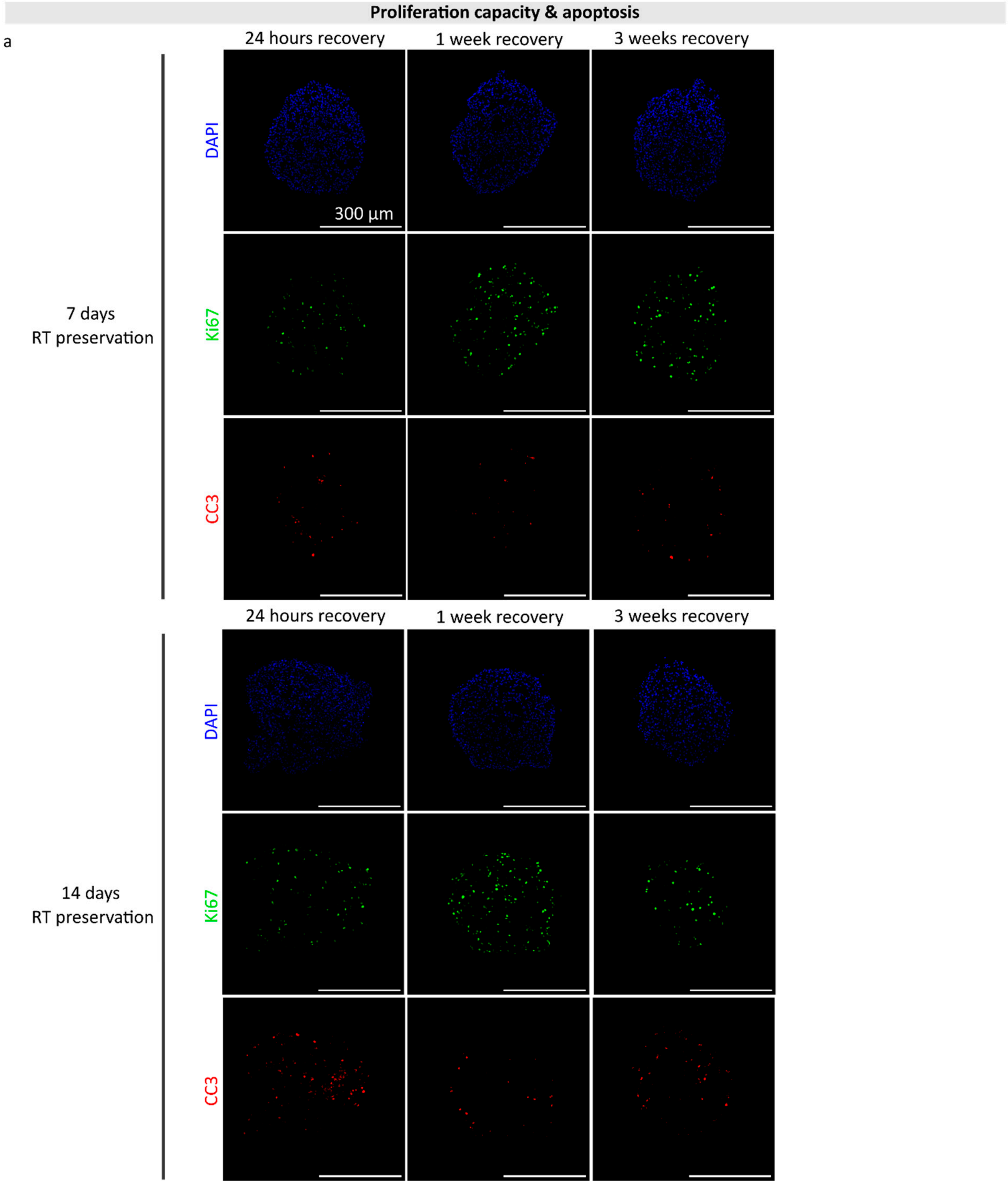

Figure S7: Proliferation capacity and apoptosis of cardioids preserved at room temperature. (**a**) Immunofluorescence images of cardioid cross sections upon room temperature (RT)-based preservation after 24 hours, one week and three weeks of recovery. Scale bar: 300 µm. DAPI: nuclear marker; Ki67: proliferation marker; CC3: apoptosis marker.

3.4.1 Immunohistochemistry for cardiomyocyte-specific marker: cardiac troponin T

We performed an additional immunostaining for cardiac troponin T to specifically evaluate cardiomyocytes, the predominant cell type in the organoids (approximately 80% of the total cell population), and to provide cell type–specific insight. Supplementary figure S8 shows the presence of cardiomyocytes in cardioids preserved at room temperature and after recovery. Normalized intensity data showed no significant differences upon preservation and recovery compared to controls. Additionally, no significant differences were detected between 7 days and 14 preservation. Overall, this data indicates no permanent alterations of cardiomyocyte presence, which is the most prominent cell type in the organoid, upon room temperature-based preservation.

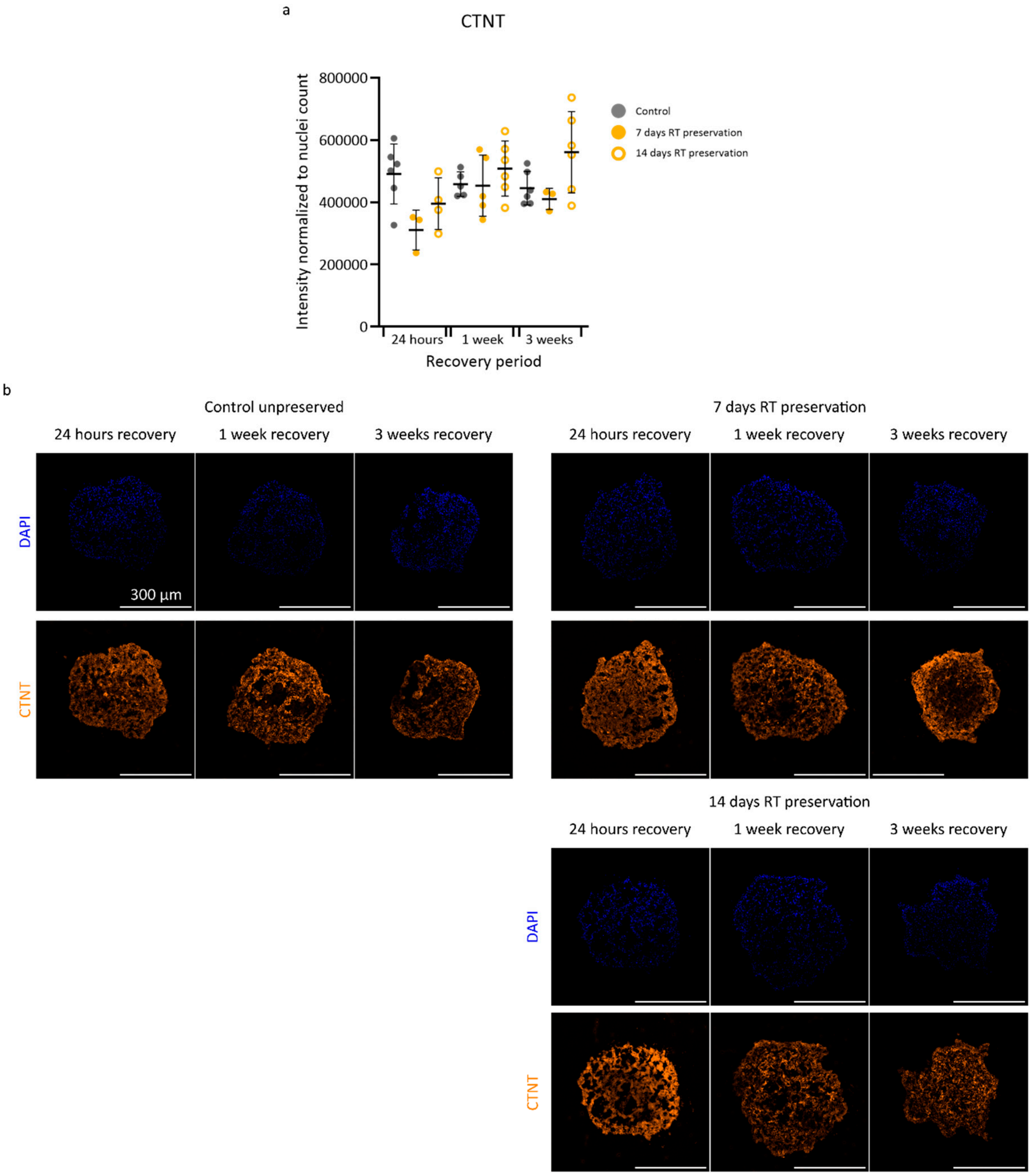

Figure S8: Presence of cardiomyocytes in cardioids preserved at room temperature. (**a**) The intensity of cardiac troponin T-positive cells, which is indicative of cardiomyocytes (N=1). The data are presented as the means ± SD, and the one-way ANOVA with Tukey's multiple comparisons test was used. *$p<0.05$. (**b**) Immunofluorescence images of cardioid cross sections upon room temperature-based preservation after 24 hours, one week and three weeks of recovery. Scale bar: 300 µm. DAPI: nuclear marker; CTNT: cardiac troponin T, cardiomyocyte-specific marker.

3.5. Correlation between cardioid size and nuclei count

Additional analysis investigating the correlation between cardioid size and nuclei count, based on DAPI staining of organoid sections, was performed. These data are included as Figure S9. Upon cryopreservation, Pearson correlation analysis revealed a significant positive correlation between cardioid size and nuclei count ($p < 0.001$, 95% confidence interval), indicating that smaller cardioids were associated with lower nuclei counts. A similar significant positive correlation was observed for room temperature–based preservation ($p = 0.0032$, 95% confidence interval). Together, these findings support the interpretation that reductions in cardioid size are at least partially associated with a decrease in cell number.

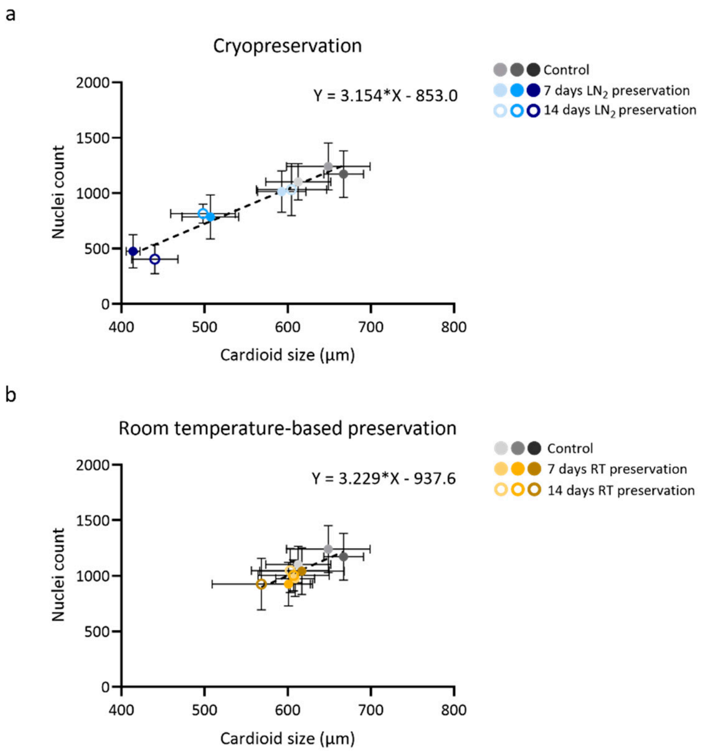

Figure S9: Correlation analysis between cardioid size and nuclei count. (**a**) Pearson correlation analysis revealed a significant positive correlation between cardioid size and nuclei count upon cryopreservation ($p<0.001$, 95% confidence interval). (**b**) A similar significant positive correlation was observed for room temperature-based preservation ($p=0.0032$, 95% confidence interval).

3.6. Normalization of beating parameters

In the original analysis, the beating parameters obtained using the MUSCLEMOTION tool were compared as raw, non-normalized values.

Additional normalization analyses were performed using the peak-to-peak time as normalization factor for the other contractile parameters. This normalization was applied to the baseline maturation data, as well as to the datasets obtained following cryopreservation and room temperature–based preservation. The normalized data are included as Supplementary Figure S10.

Following normalization of the baseline maturation data, the initially observed significant differences in contraction duration and relaxation time were no longer detected. However, a significant increase in the normalized 10-to-10 transient time between five and twenty days of maturation remained present ($p < 0.001$), although the difference between ten and twenty days maturation appeared less pronounced after normalization (Figure S10a). For the cryopreservation experiments, normalization revealed that alterations in beating behavior were in fact more pronounced than initially observed using the raw data. Significant differences were detected for contraction duration, 10-to-10 transient time, and relaxation time after both one and three weeks of recovery (Figure S10b). In addition, normalization identified a significantly increased 10-to-10 transient time following fourteen days of cryopreservation and three weeks of recovery. These findings indicate that cryopreservation-induced alterations in beating behavior persist even after correction for differences in beat frequency. In contrast, normalization of the room temperature–based preservation data showed that several initially observed differences after 24 hours and one week of recovery, particularly for contraction duration and relaxation time, were no longer significant after correction for beat rate (Figure S10c). However, after three weeks of recovery, significant alterations in these parameters became apparent. A similar trend was observed for the 10-to-10 transient time, where the initially significant effect after one week recovery disappeared following normalization, while additional significant differences became detectable after three weeks recovery.

Overall, these analyses demonstrated that normalization to beat rate refined the interpretation of the beating data and allowed a more accurate comparison of contractile parameters across experimental conditions. Importantly, the overall conclusions of the manuscript remained unchanged, namely that preservation strategies induce alterations in beating behavior reflected by changes in individual contractile parameters. However, normalization further supported the observation that room temperature–based preservation maintained beating characteristics closer to unpreserved controls after short-term recovery compared to cryopreservation.

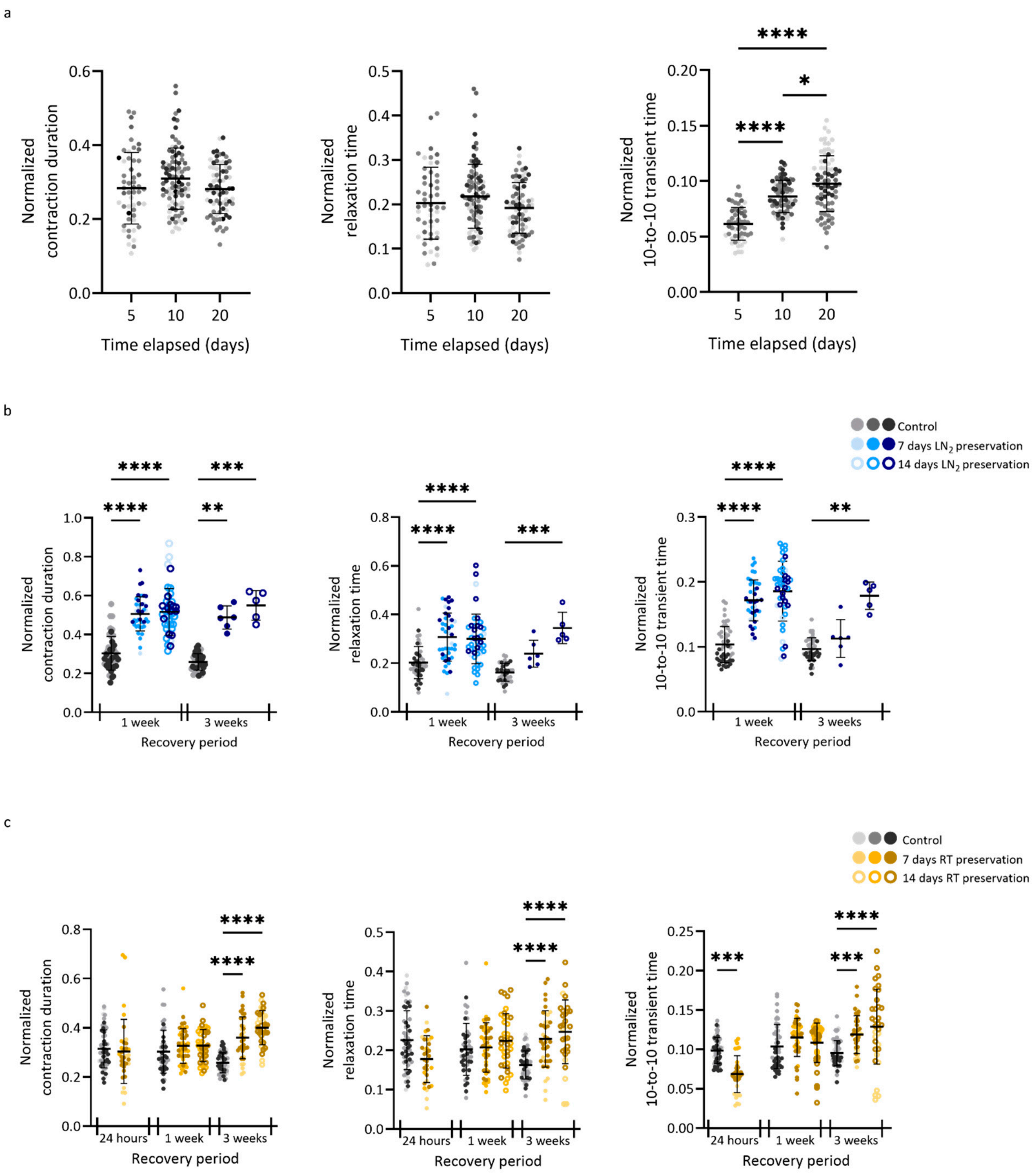

Figure S10: Normalization of beating parameters. (**a**) Baseline cardioid beating parameters, including contraction duration, relaxation time and 10-to-10 transient time, during maturation were normalized for the beat rate. The same normalization strategy was applied for beating data obtained upon cryopreservation (LN_2) (**b**) and room temperature (RT)-based preservation (**c**). Shades represent biological replicates (N=2-3). The data are presented as the means ± SDs. The Kruskal–Wallis test with Dunn's multiple comparisons test was used. *p<0.05; **p<0.01; ***p<0.005; ****p<0.001.
